# Supplementary material for: Understanding dynamics of private tuberculosis pharmacy market: a qualitative inquiry from a South Indian district
Source: BMJ Open. 2022 Jan 24;12(1):e052319. doi: 10.1136/bmjopen-2021-052319 (PMC8788189; doi:10.1136/bmjopen-2021-052319)
Supplement: Author's manuscript [file bmjopen-2021-052319.draft_revisions.pdf]

BMJ Open is committed to open peer review. As part of this commitment we make the peer review history of every article we publish publicly available.

When an article is published we post the peer reviewers' comments and the authors' responses online. We also post the versions of the paper that were used during peer review. These are the versions that the peer review comments apply to.

The versions of the paper that follow are the versions that were submitted during the peer review process. They are not the versions of record or the final published versions. They should not be cited or distributed as the published version of this manuscript.

BMJ Open is an open access journal and the full, final, typeset and author-corrected version of record of the manuscript is available on our site with no access controls, subscription charges or pay-per-view fees (http://bmjopen.bmj.com).

If you have any questions on BMJ Open's open peer review process please email info.bmjopen@bmj.com

BMJ Open

Understanding the Dynamics of Private Tuberculosis Pharmacy Market: A Qualitative Inquiry from a South Indian district

Journal:	*BMJ Open*
Manuscript ID	bmjopen-2021-052319
Article Type:	Original research
Date Submitted by the Author:	12-Apr-2021
Complete List of Authors:	Yellappa, Vijayashree; Health system transformation platform, Health service delivery<br>Bindu, Himabindu<br>Rao, Neethi<br>Narayanan, Devadasan; Institute of Public Health,
Keywords:	Tuberculosis < INFECTIOUS DISEASES, Health policy < HEALTH SERVICES ADMINISTRATION & MANAGEMENT, Organisation of health services < HEALTH SERVICES ADMINISTRATION & MANAGEMENT, Public health < INFECTIOUS DISEASES, PRIMARY CARE

SCHOLARONE™
Manuscripts

BMJ

I, the Submitting Author has the right to grant and does grant on behalf of all authors of the Work (as defined in the below author licence), an exclusive licence and/or a non-exclusive licence for contributions from authors who are: i) UK Crown employees; ii) where BMJ has agreed a CC-BY licence shall apply, and/or iii) in accordance with the terms applicable for US Federal Government officers or employees acting as part of their official duties; on a worldwide, perpetual, irrevocable, royalty-free basis to BMJ Publishing Group Ltd ("BMJ") its licensees and where the relevant Journal is co-owned by BMJ to the co-owners of the Journal, to publish the Work in this journal and any other BMJ products and to exploit all rights, as set out in our licence.

The Submitting Author accepts and understands that any supply made under these terms is made by BMJ to the Submitting Author unless you are acting as an employee on behalf of your employer or a postgraduate student of an affiliated institution which is paying any applicable article publishing charge ("APC") for Open Access articles. Where the Submitting Author wishes to make the Work available on an Open Access basis (and intends to pay the relevant APC), the terms of reuse of such Open Access shall be governed by a Creative Commons licence – details of these licences and which Creative Commons licence will apply to this Work are set out in our licence referred to above.

Other than as permitted in any relevant BMJ Author's Self Archiving Policies, I confirm this Work has not been accepted for publication elsewhere, is not being considered for publication elsewhere and does not duplicate material already published. I confirm all authors consent to publication of this Work and authorise the granting of this licence.

For peer review only

Understanding Dynamics of Private Tuberculosis Pharmacy Market: A Qualitative Inquiry from a South Indian district

Yellappa Vijayashree[1,2], *Himabindu BL[2], Neethi Rao,[2] Narayanan Devadasan[1]*

Affiliations: [1]Health System Transformation Platform, [2] Institute of Public Health, Bangalore

Corresponding author
Vijayashree Yellappa

Health System Transformation Platform

C 1 Block, ISID, 4, Institutional Area, Phase II

Vasant Kunj, New Delhi-110070

India

Telephone: 91-9008 60 1074

Email: vijayashreehy4@gmail.com

Acknowledgements: Authors would like to acknowledge Mr.Ramaiah and Ms.Amrutha for collecting the data. Authors are grateful to all respondents for their valuable time and for sharing their opinions.

Funding Statement: The work was funded by World Bank: Contract No 7165612

Authors' contributions: VY and ND conceptualised the study. VY, HB and NR coded and analysed the data. VY developed the manuscript. HB and NR refined the manuscript. All authors have read and approved the manuscript.

Conflict of interest: The authors confirm that they have no competing interests.

Ethical issues: Study received the approval from the technical committee and the institutional ethics committee of Institute of public health, Bangalore.

Abstract

Objectives: Retail private pharmacists (RPPs) are often patients' first point of contact including Tuberculosis (TB). We assessed factors influencing RPP's referral practices to Revised National TB Control programme (RNTCP) and how businesses are carried out with special reference to TB drugs.

Design: We conducted semi-structured interviews with a purposive sample of 41 RPPs, applying the principle of data saturation. Data were analysed thematically with the help of NVivo 9.

Setting: South Indian state of Karnataka, between May to October 2013.

For peer review only - http://bmjopen.bmj.com/site/about/guidelines.xhtml

Participants: 41 RPPs- 21 from urban setting and 20 from rural setting.

Results: All respondents dispensed medicines on counter, only 43% of them had pharmacy qualification. None of the RPPs had received TB related training, yet half of them knew about TB symptoms. Practice of self-referrals was common among economically poorer section who preferred purchasing medicines over-the-counter based on RPP's advice. RPPs were not aware of regulatory requirements, thus the obligatory records related to dispensing of TB drugs were not maintained. Rural RPPs tend to refer chest symptomatic to RNTCP compared to urban ones who mostly referred clients to private practitioners (PPs). TB patients' inability to buy full course of TB drugs was evident. Reciprocal relationships between RPPs, PPs and medical representatives influenced RPP's drugs stocking patterns and kickbacks. PPs wielded lot of power in this nexus.

Conclusion: Our study findings will help programme managers to develop interventions to engage RPPs in public health initiatives by taking cognisance of symbiotic relationships that exist between PPs, RPPs and medical representatives. Concurrently, there should be a strong enforcement mechanism of existing regulatory norms over the on-counter sale and records keeping.

Data availability statement

Data are not publicly available. Interested researchers may contact corresponding author for data access requests.

Key words: private pharmacy, private practitioners, RNTCP, tuberculosis, Public Private Mix, kickbacks, on-counter sale, PPM, India

Word count: 3982

Strengths and limitations of the study

- Work was undertaken to study the phenomenon naturalistically in the implementation setting, thereby providing sound empirical evidence on RPP's practices.
- Study has employed a rigorous qualitative research approach that revealed the reciprocal relationships between RPPs, Private Practitioners and medical representatives influencing RPP's drugs stocking patterns.
- Some of the initially selected pharmacists refused interviews so the data available from this small sample size may not be fully representative of the community.

For peer review only - http://bmjopen.bmj.com/site/about/guidelines.xhtml

- The study draws findings based on self-reporting by the RPPs, which may not necessarily correspond to their actual practice.
- We considered only RPPs registered with district drug controller, wherein many roadside non-registered pharmacies also dispense drugs over the counter.

Introduction

Tuberculosis (TB) is the leading infectious killer globally. Ten countries accounted for 75% of the cases, wherein India and China accounted for 39% of the global gap[1]. Though Government of India offers free quality assured TB diagnosis and treatment through Revised National TB Control Program (RNTCP)[2], more than half of TB cases are managed private practitioners (PPs) in the country[3,4]. Evidence shows that PPs rarely follow standard TB management guidelines[5,6] and thus pose a threat of severe forms of drug resistant TB, poor treatment outcomes and catastrophic out-of-pocket expenditure by the patients.

In most parts of Asia, retail private pharmacists (RPPs) are often patients' first point of contact with the health care system[7,8] and they tend to dispense cough syrups, antibiotics, anti-allergic medicines to patients with chronic cough without physician prescription and rarely refer them for TB testing[10,11,12,13]. India has about 630766 RPPs constituting an important part of the private health sector[9]. Studies have found that 83% of the surveyed RPPs received up to five prescriptions of anti-TB drugs weekly[14], a finding which was reinforced by other study which assessed the size and characteristics of private sector TB drug sales in India[15].

Evidence show that chest symptomatic who sought care from RPPs at the first instance are more likely to have long diagnostic delays[16] and nearly half of the RPPs do not refer chest symptomatic and thus contribute to delays in diagnosis and treatment[11]. It has been argued that RPPs could play an important role in the early detection of TB cases by facilitating patient pathways to TB care[10,16,17], but it is not the case now[18].

RNTCP has committed to providing free, high quality TB care to patients managed in private health sector[19]. Government of India is involving private health sector in the RNTCP through public private mix (PPM) strategy[20]. In 2012, the concept of PPM was expanded to RPPs after successful pilots in Mumbai in collaboration with Indian pharmaceutical association. Presently the governments are expected to train RPPs to identify and refer chest symptomatic to the RNTCP and provide Directly Observed Treatment Short course (DOTS) [21]. RPPs receive no payment for providing such referral services. Alongside, the Government made

For peer review only - http://bmjopen.bmj.com/site/about/guidelines.xhtml

TB a notifiable disease in 2012, and brought anti-TB medicines under designated schedule H1 drugs in 2014, which means that RPPs can sell TB drugs only against a valid prescription and are required to maintain detailed record[22].

There is dearth of literature from the developing countries on the potential of RPPs to contribute to health care[23]. Attempts at involving RPPs particularly in TB control activities have not always been successful[24,25]. To develop appropriate interventions, it is essential to understand factors that influence RPP's behaviour and how this could be changed to engage RPPs in TB control activities. With this background, we undertook a study to assess (i) RPP's referral practices linked to RNTCP (ii) stocking and dispensing patterns of TB drugs (iii) clients' TB drugs purchasing patterns and (iv) provision of kickbacks to RPPs.

This study was conducted as part of a larger research project to evaluate the results-based financing strategies for TB control in India.

Methods

Study Setting

Study was carried out in two sites in Karnataka state, India, For the rural setting, Tumkur district (population of 2.8 million) was considered since 80% of the population in the district resides in villages. For the urban setting, Tumkur city (head quarter of Tumkur district, population of 302,143) and KG Halli (population 44,000), one of the 198 administrative wards of Bangalore city was selected.

Study settings have consist of both private and public health facilities. TB services under RNTCP are provided free of cost through government facilities. Structure and functioning of RNTCP is elaborated elsewhere[26]. Patients can avail RNTCP services either directly accessing public health facilities or through referrals by PPs/RPPs.

Retail private pharmacies are privately owned and they sell drugs for profit, paid out-of-pocket by the clients. These pharmacies range from high-end big outlets staffed by qualified pharmacists to small, roadside stalls staffed by personnel without formal qualifications by utilizing the license of pharmacists who lent their certificates for money. According to regulations, minimum qualification for registration as a pharmacist is either diploma or degree in pharmacy from an institution approved by pharmacy council of India[27]. There are typically two types of private pharmacies; 'attached' are the ones which are attached to a private health facility and 'stand-alone' with no attachment to a health facility. Patients directly buy medicines from these pharmacies over-the counter with or without a valid prescription.

For peer review only - http://bmjopen.bmj.com/site/about/guidelines.xhtml

Study participants and sampling

We targeted 40 semi-structured interviews with RPPs, 20 each from rural and urban settings, applying the principle of data saturation. In Tumkur district, we randomly selected RPPs from the list maintained with district drug controller. In KG Halli, we considered all 44 pharmacies that were identified through census. Including both study sites, total of 77 pharmacies were visited. During the visits, 14 pharmacies were closed and 23 RPPs refused to participate in the study. Overall a total of 41 RPPs participated in the study (20 from rural and 21 from urban setting; 14 from KG halli and seven from Tumkur city). All except three pharmacies included in study were stand-alone stores.

Data Collection

Two members of the research team carried out the data collection from May to October 2013. Semi-structured interviews were conducted with a staff who dispensed drugs in pharmacies, irrespective of their qualification. The topic guide covered RPP's referral practices linked with RNTCP, stocking and dispensing of TB drugs, clients' TB drugs purchasing patterns and provision of kickbacks. Interview guide was translated to local language, Kannada and pilot tested before conducting the actual interviews. Written consent to participate in the study was obtained from 26 participants and the remaining opted for verbal consent. All interviews were conducted in the vicinity of pharmacies and the duration of interviews ranged from 30 to 45 minutes. All interviews were digitally recorded except four participants who refused audio recording (all from urban setting). Detailed notes were recorded from such interviews.

Data analysis

Audio-recorded interviews were translated into English and transcribed verbatim by professional transcribers. Data was managed and analysed with the support of QSR NVivo 9. Data analysis began with identifying emerging themes. Significant statements relating to the factors influencing RPP's TB management practices were identified as basic codes. VY, HB and NR devised a coding scheme jointly and this coding scheme was tested on a handful of interviews. These initial codes were then refined and organised at a broader conceptual level into themes by grouping them together[28]. Final coding framework is shown in the Table 1. In the later stages of data analysis, we explored relationships between the themes, across different categories of participants to identify patterns in the data.

Initial coding framework	Final coding framework
Qualification, age and number of years of business	**Study participants characteristics**

For peer review only - http://bmjopen.bmj.com/site/about/guidelines.xhtml

Number of health facilities present in the catchment area<br>Clients load<br>Common ailments for which drugs are dispensed on counter<br>Membership and participation in the pharmacy association	
Perception of TB<br>Awareness about RNTCP<br>Whether undergone TB training ?If, yes, where and when<br>Awareness about TB notification and other regulatory norms	**RPP's awareness about TB and RNTCP**
Factors that influence client's self-referrals to seek care from RPPs<br>Type of drugs sold over counter for chest symptoms<br>RPP's response to self-referred chest symptomatic<br>Factors influencing RPP's referrals of chest symptomatic to health providers and RNTCP	**RPP's practices linked to chest symptomatic**
Type of drugs prescribed by private practitioners for chest symptoms<br>Prescription patterns of TB drugs by PPs<br>Factors influencing RPP's stocking of TB drugs	**RPP's stocking patterns of TB drugs**
Profile of TB patients<br>Perception of problems faced by TB patients<br>Cost of TB drugs to patients<br>Factors influencing patient's purchasing of TB drugs<br>Factors influencing patient's choice of pharmacies	**Clients' drugs purchasing patterns**
Profile of the health providers who manage TB patients<br>Factors determining the health provider's choice of pharmacies and use of TB drugs<br>Provision of kickbacks to referring private practitioners	**Provision of Kickbacks to private practitioners**

Patient and public involvement

Patients and the public were not involved in the design, implementation, analysis or dissemination of the study.

Results

Participant's characteristics

Average age of study participants was 42 years and all the participants except three were male. Only 18(43%) of the participants had pharmacy qualification and others were graduates

For peer review only - http://bmjopen.bmj.com/site/about/guidelines.xhtml

in other disciplines. Work experience ranged from one to 30 years. Pharmacies were open for at least 12 hours starting from 9.00 am to 9.00 pm. Rural pharmacies had less number of PPs in the catchment area compared to urban ones, which were seemingly crowded. Nearly all RPPs were aware of professional pharmacists' associations, but only 20% of the qualified RPPs were member of these associations.

RPP's awareness about TB and RNTCP

Almost all RPPs perceived that incidence of TB is coming down and TB is no longer a problem in the community. None of the respondents had received any TB related training from RNTCP, yet half of them knew about general symptoms and mode of spread of TB. Major source of information was friends, mass media and billboards. Twelve RPPs (29%) had no idea about TB. Most RPPs knew about RNTCP, but only one RPP from the rural setting reported to have received information directly from the programme. Seven RPPs from the rural setting were aware of TB notification and they considered referrals to government hospitals to be the extent of their obligations. None of the RPPs had maintained any kind of records for dispensing TB drugs.

RPP's practices linked to chest symptomatic

RPPs reported seeing around three to four chest symptomatic per day, except six urban RPPs who reported seeing more than 20 chest symptomatic per day. Half of the respondents, mostly from the urban setting reported that they do not dispense drugs without valid prescriptions. A quote:

"If they(clients) come here directly, we do not entertain them. If they ask for medicines for small ailments, we give it. Otherwise we inform them to go to the doctor" (U_10).

Remaining half of RPPs, mostly from rural setting reported dispensing drugs over-the-counter for chest symptomatic without a prescription. They reasoned that, they do this only for patients with cold and cough as it is a common illness, as opposed to diabetes and hypertension which were considered to be serious illnesses. A narrative:

"We commonly give medicines to such people who are having cold and cough. But for Sugar and BP (hypertension), we have to give specific tablets as prescribed by doctor. Even if they (clients) ask also, we are not supposed to give like that" (R_04)

For self-refereed chest symptomatic, RPPs dispensed mix of cough syrups, anti-allergic or pain killers, out of which nine RPPs reported dispensing antibiotics to such clients. On further probing, all respondents reported that they will refer patients having cough for more than 15 days to visit nearby doctors for a thorough check-up, except one rural RPP who treated clients himself. Additionally, six respondents (four from rural and two from urban) reported

For peer review only - http://bmjopen.bmj.com/site/about/guidelines.xhtml

that they refer such patients for sputum examination and among them two (from rural area) said they would specifically refer such cases to a government facility because of the availability of free quality assured TB diagnosis there: *"I send patients to the government hospital because the results will be good"* (R_18).

Compared to the urban RPPs, rural RPPs tend to refer TB patients to the government hospital for treatment because of the availability of free TB drugs there. A quote:

"For TB patients the government hospital will provide free medicines. So they do not come outside and purchase medicines. Only patients who have consulted a private doctor, will come for AKT4 (Anti-Koch's Treatment). *But, they are very small in number"* (R_02).

When probed, majority of the respondents indicated their interest to collaborate with RNTCP, if asked for in terms of stocking DOTS drugs and referring patients for sputum examination.

RPP's stocking and dispensing of TB drugs

In total, 78% of respondents reported stocking TB drugs such as AKT3 and AKT4. RPPs mostly received TB drugs prescriptions from PPs practicing allopathy. Stocking of TB drugs was primarily based on the suggestions of PPs practicing in the catchment area. One RPP elaborated how PP's influence the stocking of drugs:

"They[doctors] *will send prescriptions. Otherwise they will write it as 'keep in stock' and send it. If a TB patient comes to our pharmacy and informs us that he will take medicines here continuously, then we will get that medicine"* (R_02).

Other factor influencing stocking of TB drugs was the promotion of certain brands by medical representatives. This phenomenon appeared to be more common and systematic in urban area where medical representatives tend to persuade PPs and RPPs to stock specific brands of TB drugs. A quote:

"*It is depending upon the doctors and medical representative's understanding. We look for two to three days. If doctor prescribes the same medicine then we decide to keep that drug"* (U_10)

Other factor influencing the stocking was the profit margins available with particular brands. Medical representatives played an important role in providing the information on profit margins and influenced RPPs choice. A quote:

"We decide by calculating profit margins of medicines. Local companies give us more margin than standard companies. Reps give us this information" (U_06)

Rural RPPs hesitated to share information about stocking and dispensing of TB drugs and the number of TB patients purchasing TB drugs compared to urban RPPs. They tend to refer patients to Government facilities since TB drugs are available free of cost there.

For peer review only - http://bmjopen.bmj.com/site/about/guidelines.xhtml

Clients' drugs purchasing patterns

Both urban and rural RPPs catered to client-load varying from 30 to 300 per day, who commonly purchased drugs for general weakness, diabetes, hypertension and respiratory tract infections. Nearly 60% of RPPs informed that, patients tend to directly visit pharmacies without consulting a doctor and others bring old prescriptions to purchase drugs over-the counter. An excerpt:

"Some of them (clients) reach here directly. They bring either the sample or the old prescription. Like this they keep renewing the old prescription without consulting doctors" (U_06).

Respondents justified the on-counter practice, as this was driven by consumer demand for fast relief of symptoms. They highlighted poverty being the key factor and patient tend to make trade-off to save money and time while seeking care. An excerpt:

"If patients go to a doctor, they have to pay consultation fee of Rs 50 to Rs 100. They are low economic class and cannot afford it. Hence they come here directly. They will get tablets and syrup for the same amount if they come here directly" (U_09)

Patients' choice of pharmacies in rural setting was based on trust, familiarity with RPPs. However in the urban setting, proximity to pharmacies, time constraint, unavailability of PPs, lack of availability of medicines in other pharmacies, relatively lower prices influenced patient's choice of pharmacy.

RPPs described TB patients as those who are in their middle age and financially poor. They estimated average cost of TB drugs per day to be 7 USD /month and could go as high as 300 USD/month if nutritional supplements, cough syrup and other antibiotics are combined together in a prescription. Some RPPs deemed the anti-TB drugs to be affordable, while an equal number of them reported TB drugs put a heavy financial strain on patients. More than 85% of respondents asserted that none of the TB patients purchased the entire course of medication at one time, instead they tend to buy drugs for few days in one go. On some occasions, patients either tend to reduce the number of drugs prescribed or purchase medicines when they have money. Urban RPPs mentioned that most patients are compelled to take loans for purchasing medicines. A quote:

"Only 30- 40% patients will buy 60% of medicines. They are mostly labour class people, doing daily wage work. They get the money only in the evening, hence they buy medicines daily" (U_05).

Provision of Kickbacks to PPs

For peer review only - http://bmjopen.bmj.com/site/about/guidelines.xhtml

RPPs having 70-100 clients per day mentioned about the provision of kickbacks to PPs, which seems to be routine in urban area, but sporadic in rural. RPPs (21%) narrated a systemic nexus that existed between RPPs, medical representatives and PPs. They estimated that PPs receive commission of about 40% from pharmaceutical companies. Few rural RPPs expressed that the provision of kickbacks to PPs is not a good practice as it has negative impact on their business.

"This is the main problem in the area. From the road till the end, 90% of the doctors are involved in this. Lot of companies are giving them some offers. If the doctor prescribes the particular medicines they get commission up to 40%" (U_06).

RPPs also reported alternate ways through which PPs made profit by owning a pharmacy attached to their clinics and got a big share in the profits. RPPs were unhappy about this arrangement as this damaged their business. A quote:

"Now a days doctors have their own medical shops. They write such prescriptions which are available with them only. They will not send the patients here, because they (doctors) will be earning commission of 30% to 40%. By chance if they take items from here, they will send it back (R_08)

Other way of earning kickbacks was to have an understanding with RPPs and PPPs tend to prescribe only such medicines that were available with that particular RPPs. A quote:

"There are doctors who have adjustments with pharmacists, and they compel patients should go to a specific pharmacy, where they get commissions" (R_10)

When RPPs were asked whether they directly pay kickbacks to PPs, all respondents denied such practice. Some regarded it as unethical with a negative impact on their reputation. One pharmacist responded:

"I have got a good name in the town. I do not give any commission to anybody. I do not get any second quality drugs neither I dispense any generic drugs" (R_02)

Discussion

Our study highlights the market dynamics that influence RPP's practices and referrals about TB patients. Study findings add value to the knowledge on the strategies involve RPPs in RNTCP. Our study showed only 43% of the respondents had pharmacy qualification and none of them had received any TB related training. RPPs were not aware about regulatory requirements related to TB, thus the obligatory records and registers required from government were not maintained. Though RPPs were aware of professional pharmacists' associations, only 20% of the qualified RPPs were member of this association. RPPs reported

For peer review only - http://bmjopen.bmj.com/site/about/guidelines.xhtml

'self-referrals' that were common among patients from economically poorer section, who preferred to purchase drugs over-the-counter based on RPP's advice. Majority of RPPs referred clients having cough more than 15 days to nearby PP. Rural RPPs were more aware of RNTCP and tend to refer TB cases to RNTCP far more compared to the urban counterparts. RPPs reported TB patients' inability to buy full course of TB treatment because of poverty. Stocking of TB drugs was based on PP's prescription patterns, marketing by medical representatives and associated profit margins. Our study demonstrates how reciprocal relationships between RPPs, PPs and medical representatives influence RPP's TB drugs stocking patterns. In general, PPs wielded substantial power in this nexus and received a significant kickbacks.

We found that half of the study participants did not have training related to pharmacy, a finding that supports the results from other studies[29,30]. This has an important implication to utilise RPP's services in TB care and thus requires attention. It is essential that personnel who dispense drugs are particularly focused for training irrespective of their qualification, since they are the interface between the community and health service. We found RPPs were willing to contribute to TB control activities if asked for, a trend reported elsewhere[31,32,33]. Therefore, a systematic policy of mapping RPPs and orienting them about RNTCP services might prove useful in timely detection of TB cases. Professional associations play a vital role in building the capacity of RPPs. However, our study found only 20% of qualified RPPs had membership with professional organisation, a finding similar to other study[34]. An exploratory research on how pharmacy association could be utilised for engaging RPPs in the TB programme would be valuable.

Our study supported the findings of studies which reported the practice of self-referrals for whom drugs were dispensed over-the-counter[35,36]. This practice was more prevalent in rural areas and RPPs there appeared to be patronised than urban RPPs[34]. RPPs who dispensed drugs over-the-counter justified the practice saying it is mostly driven by consumer demand for fast relief of symptoms, lack of time and trust with particular RPPs, as reported in other study[37,38]. Although some RPPs opined that TB drugs were affordable, patients' purchasing patterns revealed that even seemingly nominal charges could prove to be a heavy financial burden for some patients, confirming other study findings[39,40]. Such voluntary adjustments in drug purchasing by patients to reduce costs may interrupt treatment regimen aggravating the risk of drug resistance and lead to poor outcomes. Our study respondents estimated that costs of TB diagnosis and doctor's consultations costs more than the TB drugs and this could debilitate TB patients. However, data from the national surveys indicate that the majority of

For peer review only

household out-of-pocket expenditure is on drugs, which is at variance from the perceptions of study respondents[41]. This under-estimation of the financial burden of drug costs on patients may influence respondents' behaviour with regard to kickbacks, drug-pricing etc.

Our study demonstrates pharmaceutical nexus operating in the private TB drugs market. This resonates the findings from a study that explored the intense competition within the pharmaceutical industry and the key role-played by medical representatives to influence PP's prescriptions[42,43,44]. PPs not only influenced RPP's TB drugs stocking practices, but also wielded lot of power in the way RPPs carryout their routine business. A study, which analysed ways this nexus sustains itself showed that PPs compelled patients to reach particular pharmacies who provide commissions[45]. This supports the finding of a study from India which showed that PPs receive kickbacks from diagnostic laboratories and pharmacies (30%) and can earn up to INR 8000 (USD 140) from treating a single TB patient[46]. These findings point towards having a regulation that would forbid the provision of kickbacks. The state government of Maharashtra has most recently introduced a bill - Prevention of Cut Practice in Health Care Services Bill, 2017[47]. The success of this law will encourage policymakers seeking to eradicate the perverse incentives engendered through the practice of kickbacks.

None of the RPPs notified TB cases to the government nor they maintained any mandatory registers in spite of regulations to control the behaviour of private pharmacies[48], as against a systematic review finding that indicated regulation could play an important role in shaping RPP's behavior[7]. Recent efforts to regulate practices of PPs have proved to be highly contentious in Karnataka and at national levels[49]. Even though suitable laws come into force, their enforcement remains a challenge for an already stretched public health system and drug control authorities. There is a need to create a formal platform, where these two departments could collaborate to tackle these issues. Interventions to decrease the availability of TB drugs in private pharmacies and to improve referral of clients seeking TB drugs to the National TB Programme has been proven successful in South east Asian region[24]. So far there have been no such initiatives to limit the availability of TB drugs in India. Also, questions remain if such a move will be practical in India where drug stock-outs in the public health system continue to be common.

RNTCP therefore, needs to adopt multipronged interventions that combine education coupled with regulatory enforcement to engage RPPs in TB control activities. The Indian states of Gujarat and Maharashtra have experimented with the setting up of a private provider interface agency (PPIA) to facilitate engagement of RPPs in RNTCP[50]. Such PPIA may prove

For peer review only - http://bmjopen.bmj.com/site/about/guidelines.xhtml

to be a solution to structural and attitudinal impediments to effectively collaborate with private sector players. Therefore it is argued that economic rationale and the symbiotic relations that exist between PPs, medical representatives and RPPs need to be more closely scrutinized for any kind of engagement to meet public health goals[45].

We are addressing some of these issues in an intervention study in a south Indian district aimed at training RPPs to identify and refer chest symptomatic to RNTCP, opportunity to become DOTS providers and promote practice of maintaining TB drug dispensing registers[51].

Limitations of the study

The study draws findings based on self-reporting by the RPPs, which may not necessarily correspond to their actual practice. It is possible that RPPs might be skeptical about reporting actual number of TB patients due to fear of scrutiny. Although, study used stratified random sampling of rural and urban RPPs to point out differences, some selected pharmacists refused interviews. So the data available may not be fully representative of the community. We have included only such RPPs who are registered with district drug controller. But there are many roadside non-registered pharmacies, who dispense drugs over the counter.

Ethical approval

Study received approval from the institutional ethics committee of Institute of public health, Bengaluru. Personal details of participants were removed to ensure confidentiality during data transcription and analysis.

References

1. Global Tuberculosis Report. 2017. World Health Organisation. http://www.who.int/tb/publications/global_report/en/Accessed on April 17, 2018.
2. India TB Report 2018,RNTCP Annual Status Report. Ministry of Health and Family Welfare. https://tbcindia.gov.in/showfile.php?lid=3314. Accessed April 12, 2018.
3. Kapoor SK, Raman AV, Sachdeva KS, Satyanarayana S. How did the TB patients reach DOTS services in Delhi? A study of patient treatment seeking behavior. PLoS One. 2012;7(8).
4. Satyanarayana S, Nair SA, Chadha SS, Shivashankar R, Sharma G, Yadav S, et al. From where are Tuberculosis patients accessing treatment in India? results from a cross-sectional community based survey of 30 districts. PLoS One. 2011;6(9):

For peer review only - http://bmjopen.bmj.com/site/about/guidelines.xhtml

e24160. doi: 10.1371/journal.pone.0024160.

5. Satyanarayana S, Subbaraman R, Shete P, Gore G, Das J, Cattamanchi A, et al. Quality of tuberculosis care in India : a systematic review. Int J Tuberc Lung Dis. 2015; 19(7): 751-63. doi: 10.5588/ijtld.15.0186.
6. Sreeramareddy CT, Qin ZZ, Satyanarayana S, Subbaraman R, Pai M. Delays in diagnosis and treatment of pulmonary tuberculosis in India : a systematic review. Int J Tuberc Lung Dis. 2014;18(3): 255-266. doi: 10.5588/ijtld.13.0585.
7. Miller R, Goodman C. Performance of retail pharmacies in low- and middle-income Asian settings: a systematic review. Health Policy Plan. 2016;31(7): 940-953.doi:10.1093/heapol/czw007.
8. Smith F. Private local pharmacies in low- and middle-income countries: a review of interventions to enhance their role in public health. Trop Med Int Health. 2009;14(3):362–72. doi: 10.1111/j.1365-3156.2009.02232.x.
9. Global Health Observatory: Health Workforce Data by country.World Health Organization website. WHO. http://apps.who.int/gho/data/node.main.A1443?lang=en. Accessed September 27, 2016.
10. Lonnroth K, Lambregts K, Nhien DTT, Quy HT, Diwan VK. Private pharmacies and tuberculosis control: A survey of case detection skills and reported anti-tuberculosis drug dispensing in private pharmacies in Ho Chi Minh City, Vietnam. Int J Tuberc Lung Dis. 2000;4(11):1052–9.
11. Vu DH, Van Rein N, Cobelens FGJ, Nguyen TTH, Le VH, Brouwers JRBJ. Suspected tuberculosis case detection and referral in private pharmacies in Viet Nam. Int J Tuberc Lung Dis. 2012;16(12):1625–9.
12. Hurtig AK, Pande SB, Baral SC, Porter JDH, Bam DS. Anti-tuberculosis treatment in private pharmacies, Kathmandu Valley, Nepal. Int J Tuberc Lung Dis. 2000;4(8):730–6.
13. Mistry N, Rangan S, Dholakia Y, Lobo E, Shah S, Patil A. Durations and Delays in Care Seeking, Diagnosis and Treatment Initiation in Uncomplicated Pulmonary Tuberculosis Patients in Mumbai, India. PLoS One. 2016 Mar;11(3):e0152287.
14. Divakaran B, Myalil JM, Sreedharan J. Devaraya Surendranath.Sale of anti-tuberculosis drugs through private pharmacies : a cross sectional study in Kerala. India Italian journal of Publci health. 2011;8(1). DOI: http://dx.doi.org/10.2427/5639.
15. Wells WA, Ge CF, Patel N, Oh T, Gardiner E, Kimerling ME. Size and Usage Patterns of Private TB Drug Markets in the High Burden Countries. PLoS One.

For peer review only

2011;6(5):e18964. doi:10.1371/journal.pone.0018964.

16. Yellappa V, Lefvere P, Battaglioli T, Devadasan N, Van Der Styuft P. Patient Pathways to TB diagnosis and Treatment.BMC Public Health (2017) 17:635. DOI 10.1186/s12889-017-4627-7.
17. Satyanarayana S, Kwan A, Daniels B, Subbaraman R, McDowell A, Bergkvist S, et al. Use of standardised patients to assess antibiotic dispensing for tuberculosis by pharmacies in urban India: a cross-sectional study. Lancet Infect Dis. 2016 Aug;
18. Rajeswari R, Balasubramanian R, Bose MSC, Sekar L, Rahman F. Private pharmacies in tuberculosis control - A neglected link. Int J Tuberc Lung Dis. 2002;6(2):171–3.
19. Sachdeva KS, Kumar A, Dewan P, Kumar A, Satyanarayana S. New Vision for Revised National Tuberculosis Control Programme (RNTCP): Universal access - " Reaching the un-reached ." Indian J Med Res. 2012;135:690-4.
20. National Guidleines for Partnership.2014. Central TB Division, Ministry of Health and Family Welfare. https://tbcindia.gov.in/showfile.php?lid=3143. Accessed on March 3, 2016.
21. Training Module for Community Pharmacists.2013. Central TB Division. Ministry of Health and Family Welfare. https://tbcindia.gov.in/showfile.php?lid=3154. Accessed on October 3, 2017.
22. Central Drugs Standard Control Organisation Website. Minutes of the special meeting (62nd Meeting) of Drugs Technical Advisory Board held on 30th January, 2013. 2013. http://www.cdsco.nic.in/writereaddata/MinutesDTAB%2062nd.pdf. Accessed on September 5, 2017.
23. Smith F. The quality of private pharmacy services in low and middle-income countries: A systematic review. Pharm World Sci.2009;31(3):351-61.
24. Lambert ML, Delgado R, Michaux G, Vols A, Speybroeck N, Van Der Stuyft P. Collaboration between private pharmacies and national tuberculosis programme: An intervention in Bolivia. Trop Med Int Health. 2005; 10(3): 246-50.
25. Lönnroth K, Karlsson M, Lan NTN, Buu TN, Dieu TTN. Referring TB suspects from private pharmacies to the National Tuberculosis Programme: Experiences from two districts in Ho Chi Minh City, Vietnam. Int J Tuberc Lung Dis. 2003; 7(12): 1147-53.
26. Yellappa V, Lefèvre P, Battaglioli T, Narayanan D, Van Der Stuyft P. Coping with tuberculosis and directly observed treatment: a qualitative study among patients from South India. BMC Health Services Research. 2016. 16:283. DOI 10.1186/s12913-016-1545-9.

For peer review only - http://bmjopen.bmj.com/site/about/guidelines.xhtml

27. Pharmacy council of India. http://www.pci.nic.in/. Accessed on January, 2, 2018.
28. Burnard P, Gill P, Stewart K, Treasure E, Chadwick B. Analysing and presenting qualitative data. Br Dent J. 2008;204(8):429–32.
29. Basak SC, Arunkumar A KM. Community Pharmacists ’ Attitudes towards Use of Medicines in Rural India : An analysis of current situation. Int Pharm J. 2002;16(2):32–5.
30. Sabde YD, Diwan V, Saraf VS, Mahadik VK, Diwan VK, De Costa A. Mapping private pharmacies and their characteristics in Ujjain district, Central India. BMC Health Serv Res. 2011;11:351.
31. Lönnroth K, Karlsson M, Lan NTN, Buu TN, Dieu TTN. Referring TB suspects from private pharmacies to the National Tuberculosis Programme : experiences from two districts in Ho Chi Minh City , Vietnam. 2003;7:1147–53.
32. Bell CA, Eang MT, Dareth M, Rothmony E, Duncan GJ, Saini B. Provider perceptions of pharmacy-initiated tuberculosis referral services in Cambodia, 2005-2010. Int J Tuberc Lung Dis. 2012;16(8):1086–91.
33. Gharat MS, Bell CA, Ambe GT, Bell JS. Engaging community pharmacists as partners in tuberculosis control: a case study from Mumbai, India. Res Soc Adm Pharm. 2007;3(4):464–70.
34. Sabde YD, Diwan V, Saraf VS, Mahadik VK, Diwan VK, De Costa A. Mapping private pharmacies and their characteristics in Ujjain district, Central India. BMC Health Serv Res. 2011;11(1):351. doi.org/10.1186/1472-6963-11-351.
35. Basak SC, Sathyanarayana D. Evaluating medicines dispensing patterns at private community pharmacies in Tamilnadu, India. South Med Rev. 2010;3(2):27-31.
36. Brata C, Fisher C, Marjadi B, Schneider CR, Clifford RM. Factors influencing the current practice of self-medication consultations in Eastern Indonesian community pharmacies: a qualitative study. BMC Health Serv Res. 2016;16:179.
37. Rutta E, Tarimo A, Delmotte E, James I, Mwakisu S, Kasembe D, et al. Understanding private retail drug outlet dispenser knowledge and practices in tuberculosis care in Tanzania. Int J Tuberc Lung Dis. 2014;18(9):1108-13.
38. Susanne kaae, Janine Morgall Traulsen LSN. challenges to counseling customers at the pharmacy counter-why do they exist ? Res Soc Adm Pharm. 2012;8(3):253-7.
39. Tadayuki Tanimura, Ernesto Jaramillo, Diana Weil, Mario Raviglione and Knut Lo¨nnroth. Financial burden for tuberculosis patients in low- and middle-income countries: a systematic review. Eur Respir J 2014; 43: 1763–1775 | DOI:

For peer review only

For peer review only - http://bmjopen.bmj.com/site/about/guidelines.xhtml

10.1183/09031936.00193413.

40. John KR, Daley P, Kincler N, Oxlade O, Menzies D. Costs incurred by patients with pulmonary tuberculosis in rural India. Int J Tuberc Lung Dis. 2009; 13 (10): 1281-7.
41. Health in India. 2014. NSSO. Government of India. http://mospi.nic.in/sites/default/files/publication_reports/nss_rep574.pdf. Accessed on January 2, 2018.
42. Ecks S, Harper I. Public Private Mixes: the market for antituberculosis drugs in India. In: Biehl J, Petryna A. When people come first: critical issues in global health. Princeton University Press, 2013: 252–275.
43. Seeberg J. Connecting Pills and People. Med Anthropol Q. 2012;26(2):182–200.
44. Dokania AK, Dokania AK. Pharmaceutical Marketing in Rural Setting. Int J Manag Int Buisiness Stud. 2014;4(3):239–48.
45. Kamat, Vinay MN. Vinay Kamat. Soc Sci Med. 1998;47(6):779–94.
46. Yellappa V, Devadasan N, Rao N V. Evaluation of Results Based Financing Strategies for Tuberculosis care and Control in India.
47. Arun Gadre, Nilangi Sardeshpande. Cut practice in the private health care. EPW. 2017;52(48).
48. Sarda Rohit, Ladkat Nilesh, Khodade Ravikiran B. The India Pharmaceutical Industry ; Evolution Of Regulatory System And Present Scenario Introduction : Challenges Faced By Indian. International Research Journal of Pharmacy. 2012;3(6):49–55.
49. http://www.thehindu.com/news/national/karnataka/private-hospitals-up-in-arms-against-kpme-act-amendment/article19051222.ece. Accessed on December 30, 2017.
50. Sanchi Shah, Shimoni Shah, Sheela Rangan.etal. Effect of public-private interface agency in Patna and Mumbai, India: Does it alter durations and delays in care seeking for drug-sensitive pulmonary tuberculosis? Gates Open Research 2020, 4:32
51. Second Round of Impact Grant. Website of WHO-TDR: http://www.who.int/tdr/news/2015/impact-grant/en/.

For peer review only - http://bmjopen.bmj.com/site/about/guidelines.xhtml

BMJ Open

Understanding Dynamics of Private Tuberculosis Pharmacy Market: A Qualitative Inquiry from a South Indian district

Journal:	*BMJ Open*
Manuscript ID	bmjopen-2021-052319.R1
Article Type:	Original research
Date Submitted by the Author:	04-Oct-2021
Complete List of Authors:	Yellappa, Vijayashree; Institute of Public Health Bengaluru, Health service delivery<br>Bindu, Himabindu; Institute of Public Health Bengaluru, Public Health<br>Rao, Neethi; Institute of Public Health Bengaluru, Health service<br>Narayanan, Devadasan; Institute of Public Health Bengaluru, Health service
**Primary Subject Heading**:	Public health
Secondary Subject Heading:	Health policy, Health services research, Global health, Public health, Qualitative research
Keywords:	Tuberculosis < INFECTIOUS DISEASES, Health policy < HEALTH SERVICES ADMINISTRATION & MANAGEMENT, Organisation of health services < HEALTH SERVICES ADMINISTRATION & MANAGEMENT, Public health < INFECTIOUS DISEASES, PRIMARY CARE

SCHOLARONE™
Manuscripts

I, the Submitting Author has the right to grant and does grant on behalf of all authors of the Work (as defined in the below author licence), an exclusive licence and/or a non-exclusive licence for contributions from authors who are: i) UK Crown employees; ii) where BMJ has agreed a CC-BY licence shall apply, and/or iii) in accordance with the terms applicable for US Federal Government officers or employees acting as part of their official duties; on a worldwide, perpetual, irrevocable, royalty-free basis to BMJ Publishing Group Ltd ("BMJ") its licensees and where the relevant Journal is co-owned by BMJ to the co-owners of the Journal, to publish the Work in this journal and any other BMJ products and to exploit all rights, as set out in our licence.

The Submitting Author accepts and understands that any supply made under these terms is made by BMJ to the Submitting Author unless you are acting as an employee on behalf of your employer or a postgraduate student of an affiliated institution which is paying any applicable article publishing charge ("APC") for Open Access articles. Where the Submitting Author wishes to make the Work available on an Open Access basis (and intends to pay the relevant APC), the terms of reuse of such Open Access shall be governed by a Creative Commons licence – details of these licences and which Creative Commons licence will apply to this Work are set out in our licence referred to above.

Other than as permitted in any relevant BMJ Author's Self Archiving Policies, I confirm this Work has not been accepted for publication elsewhere, is not being considered for publication elsewhere and does not duplicate material already published. I confirm all authors consent to publication of this Work and authorise the granting of this licence.

For peer review only

Understanding Dynamics of Private Tuberculosis Pharmacy Market: A Qualitative Inquiry from a South Indian district

Yellappa Vijayashree[1,] *Himabindu BL[1], Neethi Rao,[1] Narayanan Devadasan[1]*

Affiliations: [1] Institute of Public Health, Bangalore

Corresponding author
Vijayashree Yellappa
Adjunct Faculty
3009, II-A Main, 17th Cross, Krishna Rajendra Rd,
Banashankari Stage II, Bengaluru, Karnataka 560070
India
Telephone: 91-9008601074
Email: vijayashreehy4@gmail.com

Ethical issues: Study received the approval from the institutional ethics committee of Institute of public health, Bangalore.

Abstract

Objectives: Retail private pharmacists (RPPs) are often patients' first point of contact including for Tuberculosis (TB) in India. We assessed the factors influencing RPP's referral of patients with chest symptoms to National TB elimination programme (NTEP) and the way business is carried out with reference to anti -TB drugs.

Design: We conducted semi-structured interviews with a purposive sample of 41 RPPs in a south Indian district between May to October 2013. Data was collected from 21 RPPs from urban area areas and 20 from rural area employing the principle of data saturation. Data were analysed thematically using NVivo 9.

Results: Both knowledge and compliance of RPPs regarding TB symptoms and regulatory requirements were found to be poor. The RPPs routinely dispensed medicines over-the-counter and less than half of respondents had pharmacy qualifications. None of them had received TB related training, yet half of them knew about TB symptoms. Practice of self-referrals was common particularly among economically poorer populations who preferred purchasing medicines over-the-counter based on RPP's advice. TB patients' inability to purchase the full course of TB drugs was conspicuous. Rural RPPs were more likely to refer patients with TB symptoms to NTEP compared to urban ones who mostly referred such clients to private practitioners (PPs). Reciprocal relationships between RPPs, PPs and medical representatives and the prevalence of kickbacks influenced RPP's drug stocking patterns. PPs wielded a lot of power in this nexus, especially in urban areas.

Conclusion: India hopes to end TB by 2025. Our study findings will help the NTEP to design policy and interventions to engage RPPs in public health initiatives by taking cognisance of symbiotic relationships and power differentials that exist between PPs, RPPs and medical representatives. Concurrently, there should be a strong enforcement mechanism for existing regulatory norms regarding over-the-counter sale and record-keeping.

For peer review only - http://bmjopen.bmj.com/site/about/guidelines.xhtml

Key words: pharmacists, private practitioners, RNTCP, NTEP, Tuberculosis, Public Private Mix, kickbacks, on-counter sale, PPM, India

Word count: 4116

Strengths and limitations of the study

- Work was undertaken to study the phenomenon naturalistically in the implementation setting, thereby providing sound empirical evidence on RPP's practices.
- Study has employed a rigorous qualitative research approach that revealed the reciprocal relationships between RPPs, PPs and medical representatives influencing RPP's drugs stocking patterns.
- Some of the initially selected pharmacists refused interviews so the data available from this study may not be fully representative of the community.
- The study draws findings based on self-reporting by RPPs, which may not necessarily correspond to their actual practice.
- We considered only RPPs registered with district drug controller, wherein many non-registered pharmacies also dispense drugs over the counter.

Introduction

Tuberculosis (TB) is the leading infectious killer globally. Ten countries accounted for 75% of the cases, wherein India and China accounted for 39% of the global gap[1]. Though Government of India offers free quality assured TB diagnosis and treatment through National TB Elimination Programme (NTEP), which was earlier known as Revised National TB Control Program[2], more than half of TB cases are managed by private practitioners (PPs) in the country[3,4]. Evidence shows that PPs rarely follow standard TB management guidelines[5,6] and thus pose a threat of severe forms of drug resistant TB.

In most parts of Asia, retail private pharmacists (RPPs) are often patients' first point of contact with the health care system[7,8] and they tend to dispense cough syrups, antibiotics, anti-allergic medicines to patients with chronic cough without physician prescription and rarely refer them for TB testing[9,10]. India has about 630766 RPPs constituting an important part of the private health sector[11] and for many patients, pharmacies may be their first point of contact, where most drugs including antibiotics, can be purchased over-the-counter[12]. Studies have found that 83% of the surveyed RPPs received up to five prescriptions of anti-TB drugs weekly[13], a finding reinforced by other study which assessed the size and characteristics of private sector anti TB drug sales in India[14].

Evidence show that chest symptomatic who sought care from RPPs at the first instance are more likely to have long diagnostic delays[15]. Nearly half of the RPPs do not refer chest symptomatic and thus contribute to delays in diagnosis and treatment[12]. Early diagnosis and treatment initiation are crucial to break the chain of transmission of TB in the community. Delays in the diagnosis increase the chances of complications and mortality. It is therefore argued that RPPs could play an important role in the early detection of TB cases by facilitating patient pathways to TB care[16,17], but it is not the case now.

NTEP has committed to providing free, high quality TB care to patients managed in private health sector through public private mix (PPM) strategy[18,19]. In 2012, the concept of PPM

For peer review only - http://bmjopen.bmj.com/site/about/guidelines.xhtml

was expanded to RPPs after successful pilots in Mumbai in collaboration with Indian pharmaceutical association. Presently the governments are expected to train RPPs to identify and refer chest symptomatic to the NTEP and provide directly observed treatment short course[20]. RPPs receive no payment for providing such referral services. Engagement of RPPs is important not only to improve TB detection and care, but also limit the abuse of antibiotics. With this background, in 2013 the government introduced the Schedule H1 as an amendment to the Drugs and Cosmetics Rule of 1945, with the intent to control rampant misuse of antibiotics through over the counter dispensing[21].This mandates the chemist to maintain a separate register where identity of the patient, contact details of the prescribing doctor and the dispensed quantity of the drug are to be recorded and maintained for at least 3 years. Further, TB was made a notifiable disease in 2012, which mandated private health players to notify TB patients either diagnosed or treated in private sector[22].

There have been studies investigating the potential of RPPs to contribute to TB care[23,24,25.] Attempts at involving RPPs particularly in TB control activities have not always been successful. There is dearth of literature from India on the potential of RPPs to contribute to TB control activities. To develop appropriate interventions, it is essential to understand factors that influence RPP's behaviour and how this could be changed to engage RPPs in TB control activities. With this background, we undertook a study to assess (i) RPP's referral practices linked to NTEP (ii) stocking and dispensing patterns of anti -TB drugs (iii) clients' TB drugs purchasing patterns and (iv) explore the provision of kickbacks to RPPs, if any.

This study was conducted as part of a larger research project to evaluate the results-based financing strategies for TB control in India.

Methods

Study Setting

Study was carried out in two sites in Karnataka state, India, For the rural setting, Tumkur district (population of 2.8 million) was considered since 80% of the population in the district resides in villages. For the urban setting, Tumkur city (head quarter of Tumkur district, population of 302,143) and KG Halli (population 44,000), one of the 198 administrative wards of Bangalore city was selected.

Study settings consist of both private and public health facilities. TB services under NTEP are provided free of cost through government facilities. Structure and functioning of NTEP is elaborated elsewhere[26]. Patients can avail NTEP services either directly accessing public health facilities or through referrals by PPs/RPPs.

Retail private pharmacies are privately owned and they sell drugs for profit, paid out-of-pocket by the clients. These pharmacies range from high-end big outlets staffed by qualified pharmacists to small, roadside stalls staffed by personnel without formal qualifications by utilizing the license of pharmacists who lent their certificates for money. According to regulations, minimum qualification for registration as a pharmacist is either diploma or degree in pharmacy from an institution approved by pharmacy council of India[27]. There are typically two types of private pharmacies; 'attached' are the ones which are attached to a private health facility and 'stand-alone' with no attachment to a health facility. Patients directly buy medicines from these pharmacies over-the counter with or without a valid prescription.

For peer review only - http://bmjopen.bmj.com/site/about/guidelines.xhtml

Study participants and sampling

We targeted 40 semi-structured interviews purposively with RPPs, 20 each from rural and urban settings, applying the principle of data saturation. In Tumkur district, we randomly selected RPPs from the list maintained with district drug controller. In KG Halli, we considered all 44 pharmacies that were identified through census. Including both study sites, total of 77 pharmacies were visited. During the visits, 14 pharmacies were closed and 23 RPPs refused to participate in the study. Overall a total of 41 RPPs participated in the study (20 from rural and 21 from urban setting; 14 from KG halli and seven from Tumkur city). All except three pharmacies included in study were stand-alone stores.

Data Collection

Data collection happened May to October 2013. Semi-structured interviews were conducted with a staff who dispensed drugs in pharmacies, irrespective of their qualification. The topic guide covered RPP's referral practices linked with NTEP, stocking and dispensing of TB drugs, clients' TB drugs purchasing patterns and provision of kickbacks. Interview guide was translated to local language, Kannada and pilot tested before conducting the actual interviews. Information brochure was shared with participants and the objectives of the research was explained. An appointment was sought and interviews were conducted in the vicinity of pharmacies. Duration of interviews ranged from 30 to 45 minutes. All interviews were digitally recorded except four participants who refused audio recording (all from urban setting). Detailed notes were recorded from such interviews.

Data analysis

Audio-recorded interviews were translated into English and transcribed verbatim by professional transcribers. Data was managed and analysed with the support of QSR NVivo 9. We conducted a thematic analysis[28,29]. We combined deductive and inductive approaches to analyse the data. The deductive approach was based on the research questions and new themes emerging from the data were included (inductive approach)[30]. Significant statements relating to the factors influencing RPP's TB management practices were identified as basic codes. VY, HB and NR devised a coding scheme jointly and this coding scheme was tested on a handful of interviews. These initial codes were then refined and organised at a broader conceptual level into themes by grouping them together[28]. Final coding framework is shown in the Table 1. In the later stages of data analysis, we explored relationships between the themes, across different categories of participants to identify patterns in the data. To increase the internal validity of the analysis, the coding scheme, the memos and the emerging themes were regularly discussed among the authors. Figure 1 represents the different themes emerged from the data.

Table 1: Coding Framework

Initial coding framework	Final coding framework
Qualification, age and number of years of business<br>Number of health facilities present in the catchment area<br>Clients load	**Study participants characteristics**

For peer review only - http://bmjopen.bmj.com/site/about/guidelines.xhtml

Common ailments for which drugs are dispensed on counter<br>Membership and participation in the pharmacy association	
Perception of TB<br>Awareness about NTEP<br>Whether undergone TB training ?If, yes, where and when<br>Awareness about TB notification and other regulatory norms	**RPP's awareness about TB and** NTEP
Factors that influence client's self-referrals to seek care from RPPs<br>Type of drugs sold over counter for chest symptoms<br>RPP's response to self-referred chest symptomatic<br>Factors influencing RPP's referrals of chest symptomatic to health providers and NTEP	**RPP's practices linked to chest symptomatic**
Type of drugs prescribed by private practitioners for chest symptoms<br>Prescription patterns of TB drugs by PPs<br>Factors influencing RPP's stocking of TB drugs	**RPP's stocking patterns of TB drugs**
Profile of TB patients<br>Perception of problems faced by TB patients<br>Cost of TB drugs to patients<br>Factors influencing patient's purchasing of TB drugs<br>Factors influencing patient's choice of pharmacies	**Clients' drugs purchasing patterns**
Profile of the health providers who manage TB patients<br>Factors determining the health provider's choice of pharmacies and use of TB drugs<br>Provision of kickbacks to referring private practitioners	**Provision of Kickbacks to private practitioners**

Patient and public involvement: Patients and the public were not involved in the design, implementation, analysis or dissemination of the study.

Results

Participant's characteristics

Average age of study participants was 42 years. Only 18(43%) of the participants had pharmacy qualification and others were graduates in other disciplines. Pharmacies were open for at least 12 hours starting from 9.00 am to 9.00 pm. Rural pharmacies had less number of PPs in the catchment area compared to urban ones, which were seemingly crowded. Nearly all RPPs were aware of professional pharmacists' associations, but only 20% of the qualified RPPs were member of these associations. Details of RPPs characteristics are provided in table 2.

For peer review only - http://bmjopen.bmj.com/site/about/guidelines.xhtml

Table 2- Demographic data of the RPPs

Characteristic	Rural	Urban
	Gender	
Male	22	16
Female	1	2
	Age	
18-29	6	3
30-49	15	10
50 and above	2	4
	Number of years working at the pharmacy	
0-5	4	4
6-10	9	9
11-20	6	3
>20	2	0
Not available	2	0
	Approximate number of customers per day	
0-50	8	8
51-100	8	6
>100	3	2
Not available	4	1
	Approximate number of patients with complaint of cough per day	
0	1	1
1-15	5	9
16-30	2	1
>30	0	1
Not available	15	5

***RPP's awareness about TB and** NTEP*

Almost all RPPs perceived that incidence of TB is coming down and it is no longer a problem in the community. None of the respondents had received any TB related training from NTEP, yet half of them knew about general symptoms and mode of spread of TB. Major source of information was friends, mass media and billboards. Twelve RPPs (29%) had no idea about TB. Most RPPs knew about NTEP, but only one RPP from the rural setting reported to have received information directly from the programme. Seven RPPs from the rural setting were aware of TB notification and they considered referrals to government hospitals to be the extent of their obligations. None of the RPPs had maintained any kind of records for dispensing TB drugs.

RPP's practices in managing patients with chest symptoms

RPPs reported seeing around three to four chest symptomatic per day, except six urban RPPs who reported seeing more than 20 chest symptomatic per day. Half of the respondents, mostly from urban setting reported that they do not dispense drugs without valid prescriptions. A quote:

For peer review only - http://bmjopen.bmj.com/site/about/guidelines.xhtml

"If they(clients) come here directly, we do not entertain them. If they ask for medicines for small ailments, we give it. Otherwise we inform them to go to the doctor" (U_10).

Remaining half of RPPs, mostly from rural setting reported dispensing drugs over-the-counter for chest symptomatic without a prescription. They reasoned that, they do this only for patients with cold and cough as it is a common illness, as opposed to diabetes and hypertension which were considered to be serious illnesses. A narrative:

"We commonly give medicines to such people who are having cold and cough. But for Sugar and BP (hypertension), we have to give specific tablets as prescribed by doctor. Even if they (clients) ask also, we are not supposed to give like that" (R_04)

For self-refereed chest symptomatic, RPPs dispensed mix of cough syrups, anti-allergic or pain killers, out of which nine RPPs reported dispensing antibiotics to such clients. On further probing, all respondents reported that they will refer patients having cough for more than 15 days to visit nearby doctors for a thorough check-up, except one rural RPP who treated clients himself. Additionally, six respondents (four from rural and two from urban) reported that they refer such patients for sputum examination and among them two (from rural area) said they would specifically refer such cases to a government facility because of the availability of free quality assured TB diagnosis there: *"I send patients to the government hospital because the results will be good"* (R_18).

"For TB patients the government hospital will provide free medicines. So they do not come outside and purchase medicines. Only patients who have consulted a private doctor, will come for AKT4 (Anti-Koch's Treatment). *But, they are very small in number"* (R_02).

When probed, majority of the respondents indicated their interest to collaborate with NTEP, if asked for in terms of stocking DOTS drugs and referring patients for sputum examination.

RPP's stocking and dispensing of TB drugs

In total, 78% of respondents reported stocking TB drugs such as AKT3 and AKT4. Stocking of TB drugs was primarily based on the suggestions of PPs practicing in the catchment area. One RPP elaborated how PP's influence the stocking of drugs:

"They[doctors] *will send prescriptions. Otherwise they will write it as 'keep in stock' and send it. If a TB patient comes to our pharmacy and informs us that he will take medicines here continuously, then we will get that medicine" (*R_02).

Other factor influencing stocking of TB drugs was the promotion of certain brands by medical representatives to provide information on profit margins. This heavily influenced RPPs stocking choices. This phenomenon appeared to be more common and systematic in urban area where medical representatives tend to persuade PPs and RPPs to stock specific brands of TB drugs. A quote:

"*It is depending upon the doctors and medical representative's understanding. We look for two to three days. If doctor prescribes the same medicine then we decide to keep that drug"* (U_10)

"We decide by calculating profit margins of medicines. Local companies give us more margin than standard companies. Reps give us this information" (U_06)

Rural RPPs hesitated to share information about stocking and dispensing of TB drugs and the number of TB patients purchasing TB drugs compared to urban RPPs. They tend to refer patients to Government facilities since TB drugs are available free of cost there.

For peer review only - http://bmjopen.bmj.com/site/about/guidelines.xhtml

Clients' drugs purchasing patterns

Both urban and rural RPPs catered to client-load varying from 30 to 300 per day, who commonly purchased drugs for general weakness, diabetes, hypertension and respiratory tract infections. Nearly 60% of RPPs informed that, patients tend to directly visit pharmacies without consulting a doctor and others bring old prescriptions to purchase drugs over-the counter. Respondents justified the on-counter practice, as this was driven by consumer demand for fast relief of symptoms. They highlighted poverty being the key factor and patient tend to make trade-off to save money and time while seeking care. An excerpt:

"If patients go to a doctor, they have to pay consultation fee of Rs 50 to Rs 100. They are low economic class and cannot afford it. Hence they come here directly. They will get tablets and syrup for the same amount if they come here directly" (U_09)

Patients' choice of pharmacies in rural setting was based on trust, familiarity with RPPs. However in the urban setting, proximity to pharmacies, time constraint, unavailability of PPs, lack of availability of medicines in other pharmacies, relatively lower prices influenced patient's choice of pharmacy.

RPPs described TB patients as those who are in their middle age and financially poor. They estimated average cost of TB drugs per day to be 7 USD /month and could go as high as 300 USD/month if nutritional supplements, cough syrup and other antibiotics are combined together in a prescription. Some RPPs deemed the anti-TB drugs to be affordable, while an equal number of them reported TB drugs put a heavy financial strain on patients. More than 85% of respondents asserted that none of the TB patients purchased the entire course of medication at one time, instead they tend to buy drugs for few days in one go. On some occasions, patients either tend to reduce the number of drugs prescribed or purchase medicines when they have money. Urban RPPs mentioned that most patients are compelled to take loans for purchasing medicines. A quote:

"Only 30- 40% patients will buy 60% of medicines. They are mostly labour class people, doing daily wage work. They get the money only in the evening, hence they buy medicines daily" (U_05).

Provision of Kickbacks to PPs

RPPs having 70-100 clients per day mentioned about the provision of kickbacks to PPs seems to be routine in urban area, but sporadic in rural. RPPs (21%) narrated a systemic nexus that existed between RPPs, medical representatives and PPs. They estimated that PPs receive commission of about 40% from pharmaceutical companies. Few rural RPPs expressed that the provision of kickbacks to PPs is not a good practice as it has negative impact on their business.

"This is the main problem in the area. From the road till the end, 90% of the doctors are involved in this. Lot of companies are giving them some offers. If the doctor prescribes the particular medicines they get commission up to 40%" (U_06).

RPPs also reported alternate ways through which PPs made profit by owning a pharmacy attached to their clinics and got a big share in the profits. RPPs were unhappy about this arrangement as this damaged their business. A quote:

"Now a days doctors have their own medical shops. They write such prescriptions which are available with them only. They will not send the patients here, because they (doctors) will be

For peer review only - http://bmjopen.bmj.com/site/about/guidelines.xhtml

earning commission of 30% to 40%. By chance if they take items from here, they will send it back (R_08)

Other way of earning kickbacks was to have an understanding with RPPs and PPPs tend to prescribe only such medicines that were available with that particular RPPs. A quote:

"There are doctors who have adjustments with pharmacists, and they compel patients should go to a specific pharmacy, where they get commissions" (R_10)

When RPPs were asked whether they directly pay kickbacks to PPs, all respondents denied such practice. Some regarded it as unethical with a negative impact on their reputation.

Discussion

Our study highlights the market dynamics that influence RPP's referrals patterns about presumptive TB patients and stocking patterns of anti-TB drugs. Study findings add value to the knowledge on the strategies to involve RPPs in the NTEP. Our study showed only 43% of the respondents had pharmacy qualification and none of them had received any TB related training. RPPs reported they were not aware about regulatory requirements related to TB, thus the obligatory records and registers required from government were not maintained. Though RPPs were aware of professional pharmacists' associations, only 20% of the qualified RPPs were member of this association. RPPs reported 'self-referrals' were common among patients from economically poorer section, who preferred to purchase drugs over-the-counter based on RPP's advice. Majority of RPPs referred clients having cough more than 15 days to nearby PPs. Rural RPPs were more aware of NTEP and tend to refer TB cases to NTEP far more compared to the urban counterparts. RPPs reported TB patients' inability to buy full course of TB treatment because of poverty. Our study demonstrates how reciprocal relationships between RPPs, PPs and medical representatives influence RPP's anti - TB drugs stocking patterns. In general, PPs wielded substantial power in this nexus and received a significant kickbacks.

We found that half of the study participants did not have training related to pharmacy, a finding that supports the results from other study[31]. Hence, it is essential that personnel who dispense drugs should be particularly be targeted for public health training irrespective of their qualification. We found RPPs were willing to contribute to TB control activities if asked for, a trend reported elsewhere[32,33,34]. Therefore, a systematic policy of mapping RPPs and orienting them about NTEP services might prove useful in timely detection of TB cases. Professional associations play a vital role in building the capacity of RPPs. However, our study found only 20% of qualified RPPs had membership with professional organisation, a finding similar to other study[35]. A policy or an incentive to encourage RPPs to join Indian Pharmaceutical Associations, wherein they could be systematically trained.

Our study supported the findings of studies which reported the practice of self-referrals for whom drugs were dispensed over-the-counter[36,37], which was more prevalent in rural areas. We found rural RPPs were more patronised than urban ones. RPPs who dispensed drugs over-the-counter justified the practice stating it is mostly driven by consumer demand for fast relief of symptoms as reported in other study[38,39]. Although some RPPs opined that TB drugs were affordable, patients' purchasing patterns revealed that even seemingly nominal charges could prove to be a heavy financial burden for some patients, confirming other study findings[40,41]. Such voluntary adjustments in drug purchasing by patients to reduce costs may

For peer review only - http://bmjopen.bmj.com/site/about/guidelines.xhtml

aggravate the risk of drug resistance and lead to poor outcomes. Our study respondents estimated that costs of TB diagnosis and doctor's consultations costs more than the TB drugs and this could debilitate TB patients. However, data from the national surveys indicate that the majority of household out-of-pocket expenditure is on drugs, which is at variance from the perceptions of study respondents[42]. This under-estimation of the financial burden due to drug costs may influence respondents' behaviour with regard to kickbacks, drug-pricing etc.

Our study demonstrates pharmaceutical nexus operating in the private anti-TB drugs market. Opinions of pharma company representatives influenced stocking and sale of anti-TB drugs. This resonates the findings from a study that explored the intense competition within the pharmaceutical industry and the key role-played by medical representatives to influence PP's prescriptions[43,44,45]. This finding hints at the possibility exploring a business model for subsidising private anti-TB drugs with pharma industries and also training pharma company representatives about the NTEP provisions.

PPs not only influenced RPP's TB drugs stocking practices, but also wielded lot of power, by forcing RPPs to stock medicines of their choice. This supports the finding of a study from India which showed that PPs receive kickbacks from laboratories and pharmacies (30%)[46]. This points towards formulating a regulation that forbids practise of kickbacks. Government of Maharashtra has introduced a bill - Prevention of Cut Practice in Health Care Services Bill, 2017[47]. Evaluation of the effectiveness of this law would be helpful to curb the perverse incentives engendered through the practice of kickbacks.

None of the RPPs notified TB cases nor they maintained any mandatory registers in spite of regulatory requirement. Data sharing on anti-TB drugs from pharmacies in India continues to be poor[48]. There are mixed findings about the effectiveness of Schedule H1 regulation. Some studies have shown that it has minimized on counter dispensing of first-line anti-TB drugs[49,50], but another study from south Indian city reported continued irrational dispensing of antibiotics by private pharmacies[51]. Even though suitable laws come into force, their enforcement remains a challenge for an already stretched public health system and drug control authorities.

RPPs are not optimally represented in the national policy discussions in spite of the role played by them as an interface. Though NTEP's National strategic plan[18] though mentions about the need for engaging chemists, modalities for operationalising this vision is lacking. It is imperative that the symbiotic relations existing between PPs, medical representatives and RPPs should be closely scrutinized for any kind of engagement to meet public health goals. States of Gujarat and Maharashtra have experimented with the setting up of a private provider interface agency to facilitate engagement of RPPs in the NTEP[52]. Investing in public provider support agency[19] that respond to pharmacists' profit making needs while promoting the optimal delivery of health care services need to be prioritised.

NTEP thus needs to adopt multipronged interventions that combine education coupled with regulatory enforcement to engage RPPs in TB control activities. We have addressed some of these issues in an intervention study conducted in a south Indian district [53].

Limitations of the study

Study draws findings based on self-reporting by the RPPs, which may not necessarily correspond to their actual practice. It is possible that RPPs might be sceptical about reporting

For peer review only - http://bmjopen.bmj.com/site/about/guidelines.xhtml

actual number of TB patients due to fear of scrutiny. Alternatively, this discrepancy could be attributed to socially desirable responses by the participants. To overcome this, we used various approaches including assuring the RPPs of anonymity and confidentiality of the information collected, indirect questioning, probing where RPPs were not particularly forthcoming with the information, amongst others. However, we acknowledge the potential for eliciting politically correct response exists and therefore the potential for over/underestimation of the extent of these issues.
Some selected pharmacists refused interviews. So the data available may not be fully representative of the community. We have included only such RPPs who are registered with district drug controller. But there are many non-registered pharmacies, who dispense drugs over the counter.

Acknowledgements: Authors would like to acknowledge Mr.Ramaiah and Ms.Amrutha for facilitating data collection.

Authors' contributions: VY and ND conceptualised the study. VY collected the data and developed the manuscript. VY, HB and NR coded and analysed the data. HB and NR contributed to the manuscript. All authors have read and approved the manuscript.

Funding Statement: The work was funded by World Bank: Contract No 7165612

Conflict of interest: The authors confirm that they have no competing interests.

Patient consent for publication Not required.

Ethics approval
Study received approval from the institutional ethics committee of Institute of public health, Bengaluru. After explaining the confidentiality in local language, written consent to participate in the study was obtained from 26 participants and the remaining opted for verbal consent. Authorization for audio recording the interviews was also sought. Personal details of participants were removed to ensure confidentiality during data transcription and analysis and the audio files were anonymised. The NVivo database was password protected and was only accessible to the research team.

Data sharing statement
Data availability statement Data are available upon reasonable request.

Figure 1: Different themes emerged from the data.

References

1. Global Tuberculosis Report. 2020. World Health Organisation. https://apps.who.int/iris/bitstream/handle/10665/337538/9789240016095-eng.pdf.

For peer review only - http://bmjopen.bmj.com/site/about/guidelines.xhtml

Accessed on August 17, 2021.
2. India TB Report 2018,RNTCP Annual Status Report. Ministry of Health and Family Welfare. https://tbcindia.gov.in/showfile.php?lid=3314. Accessed April 12, 2018.
3. Kapoor SK, Raman AV, Sachdeva KS, Satyanarayana S. How did the TB patients reach DOTS services in Delhi? A study of patient treatment seeking behavior. PLoS One. 2012;7(8).
4. Satyanarayana S, Nair SA, Chadha SS, Shivashankar R, Sharma G, Yadav S, et al. From where are Tuberculosis patients accessing treatment in India? results from a cross-sectional community based survey of 30 districts. PLoS One. 2011;6(9): e24160. doi: 10.1371/journal.pone.0024160.
5. Satyanarayana S, Subbaraman R, Shete P, Gore G, Das J, Cattamanchi A, et al. Quality of tuberculosis care in India : a systematic review. Int J Tuberc Lung Dis. 2015; 19(7): 751-63. doi: 10.5588/ijtld.15.0186.
6. Deo, S., Jindal, P. & Papineni, S. Integrating Xpert MTB/RIF for TB diagnosis in the private sector: evidence from large-scale pilots in Patna and Mumbai, India. *BMC Infect Dis*.2021; 21 (123)
7. Miller R, Goodman C. Performance of retail pharmacies in low- and middle-income Asian settings: a systematic review. Health Policy Plan. 2016;31(7): 940-953.doi:10.1093/heapol/czw007.
8. Smith F. Private local pharmacies in low- and middle-income countries: a review of interventions to enhance their role in public health. Trop Med Int Health. 2009;14(3):362–72. doi: 10.1111/j.1365-3156.2009.02232.x.
9. Vu DH, Van Rein N, Cobelens FGJ, Nguyen TTH, Le VH, Brouwers JRBJ. Suspected tuberculosis case detection and referral in private pharmacies in Viet Nam. Int J Tuberc Lung Dis. 2012;16(12):1625–9.
10. Mistry N, Rangan S, Dholakia Y, Lobo E, Shah S, Patil A. Durations and Delays in Care Seeking, Diagnosis and Treatment Initiation in Uncomplicated Pulmonary Tuberculosis Patients in Mumbai, India. PLoS One. 2016 Mar;11(3):e0152287.
11. Global Health Observatory: Health Workforce Data by country.World Health Organization website. WHO. http://apps.who.int/gho/data/node.main.A1443?lang=en. Accessed September 27, 2016.
12. Revathy R, Sahu SK, Rehman T. Status of mandatory tuberculosis case notification among pharmacy outlets in urban Puducherry: A cross-sectional descriptive study. Indian J Community Med 2021;46:344-5
13. Divakaran B, Myalil JM, Sreedharan J. Devaraya Surendranath.Sale of anti-tuberculosis drugs through private pharmacies : a cross sectional study in Kerala. India Italian journal of Publci health. 2011;8(1). DOI: http://dx.doi.org/10.2427/5639.
14. Wells WA, Ge CF, Patel N, Oh T, Gardiner E, Kimerling ME. Size and Usage Patterns of Private TB Drug Markets in the High Burden Countries. PLoS One. 2011;6(5):e18964. doi:10.1371/journal.pone.0018964.
15. Yellappa V, Lefvere P, Battaglioli T, Devadasan N, Van Der Styuft P. Patient Pathways to TB diagnosis and Treatment.BMC Public Health (2017) 17:635. DOI 10.1186/s12889-017-4627-7.
16. Satyanarayana S, Kwan A, Daniels B, Subbaraman R, McDowell A, Bergkvist S, et

For peer review only - http://bmjopen.bmj.com/site/about/guidelines.xhtml

al. Use of standardised patients to assess antibiotic dispensing for tuberculosis by pharmacies in urban India: a cross-sectional study. Lancet Infect Dis. 2016 ;16(11):1261-1268.
17. Rajeswari R, Balasubramanian R, Bose MSC, Sekar L, Rahman F. Private pharmacies in tuberculosis control - A neglected link. Int J Tuberc Lung Dis. 2002;6(2):171–3.
18. National Strategic Plan to End TB in India 2020-25. Central TB Division, Ministry of Health and Family. Welfare.https://tbcindia.gov.in/index1.php?lang=1&level=2&sublinkid=5507&lid=3528. Accessed on May 2021.
19. Guidance Document on Partnerships. 2019. Revised National Tuberculosis Control Programme. Central TB Division. Ministry of Health and Family Welfare. https://tbcindia.gov.in/showfile.php?lid=3456. Accessed on October 17 2020.
20. Training Module for Community Pharmacists.2013. Central TB Division. Ministry of Health and Family Welfare. https://tbcindia.gov.in/showfile.php?lid=3154. Accessed on October 3, 2017.
21. Hazra A. Schedule H1: Hope or hype?. Indian J Pharmacol 2014;46:361-2
22. Mandatory TB notification Gazette for private practitioners, chemists and public health staff. https://tbcindia.gov.in/WriteReadData/l892s/5329920697FAQs%20on%20Mandatory%20TB%20notification%20Gazette%20English.pdf.
23. Daftary A, Satyanarayana S, Jha N, *et al.* Can community pharmacists improve tuberculosis case finding? A mixed methods intervention study in India. *BMJ Global Health* 2019;4:e001417.
24. Konduri, N., Delmotte, E. & Rutta, E. Engagement of the private pharmaceutical sector for TB control: rhetoric or reality?. *J of Pharm Policy and Pract* 10, 6 (2017).
25. Miller R, Goodman C. Quality of tuberculosis care by pharmacies in low- and middle-income countries: Gaps and opportunities. *J Clin Tuberc Other Mycobact Dis*. 2019;18:100135. Published 2019 Dec 2. doi:10.1016/j.jctube.2019.100135.
26. Yellappa V, Lefèvre P, Battaglioli T, Narayanan D, Van Der Stuyft P. Coping with tuberculosis and directly observed treatment: a qualitative study among patients from South India. BMC Health Services Research. 2016. 16:283. DOI 10.1186/s12913-016-1545-9.
27. Pharmacy council of India. http://www.pci.nic.in/.
28. Boyatzis RE. Transforming qualitative information: thematic analysis and code development. Thousand Oaks CA: Sage; 1998.
29. Mq P. Qualitative Research and Evaluation Methods. 3rd ed. Thousand Oaks CA: Sage; 2002.
30. Burnard P, Gill P, Stewart K, Treasure E, Chadwick B. Analysing and presenting qualitative data. Br Dent J. 2008;204(8):429–32.
31. Basak SC, Arunkumar A KM. Community Pharmacists ' Attitudes towards Use of Medicines in Rural India : An analysis of current situation. Int Pharm J. 2002;16(2):32–5.
32. Sabde YD, Diwan V, Saraf VS, Mahadik VK, Diwan VK, De Costa A. Mapping private pharmacies and their characteristics in Ujjain district, Central India. BMC

For peer review only - http://bmjopen.bmj.com/site/about/guidelines.xhtml

Health Serv Res. 2011;11:351.
33. Bell CA, Eang MT, Dareth M, Rothmony E, Duncan GJ, Saini B. Provider perceptions of pharmacy-initiated tuberculosis referral services in Cambodia, 2005-2010. Int J Tuberc Lung Dis. 2012;16(8):1086–91.
34. Gharat MS, Bell CA, Ambe GT, Bell JS. Engaging community pharmacists as partners in tuberculosis control: a case study from Mumbai, India. Res Soc Adm Pharm. 2007;3(4):464–70.
35. Sabde YD, Diwan V, Saraf VS, Mahadik VK, Diwan VK, De Costa A. Mapping private pharmacies and their characteristics in Ujjain district, Central India. BMC Health Serv Res. 2011;11(1):351. doi.org/10.1186/1472-6963-11-351.
36. Basak SC, Sathyanarayana D. Evaluating medicines dispensing patterns at private community pharmacies in Tamilnadu, India. South Med Rev. 2010;3(2):27-31.
37. Brata C, Fisher C, Marjadi B, Schneider CR, Clifford RM. Factors influencing the current practice of self-medication consultations in Eastern Indonesian community pharmacies: a qualitative study. BMC Health Serv Res. 2016;16:179.
38. Rutta E, Tarimo A, Delmotte E, James I, Mwakisu S, Kasembe D, et al. Understanding private retail drug outlet dispenser knowledge and practices in tuberculosis care in Tanzania. Int J Tuberc Lung Dis. 2014;18(9):1108-13.
39. Susanne kaae, Janine Morgall Traulsen LSN. challenges to counseling customers at the pharmacy counter-why do they exist ? Res Soc Adm Pharm. 2012;8(3):253-7.
40. Tadayuki Tanimura, Ernesto Jaramillo, Diana Weil, Mario Raviglione and Knut Lo¨nnroth. Financial burden for tuberculosis patients in low- and middle-income countries: a systematic review. Eur Respir J 2014; 43: 1763–1775 | DOI: 10.1183/09031936.00193413.
41. John KR, Daley P, Kincler N, Oxlade O, Menzies D. Costs incurred by patients with pulmonary tuberculosis in rural India. Int J Tuberc Lung Dis. 2009; 13 (10): 1281-7.
42. Health in India. 2014. NSSO. Government of India. http://mospi.nic.in/sites/default/files/publication_reports/nss_rep574.pdf. Accessed on January 2, 2018.
43. Ecks S, Harper I. Public Private Mixes: the market for antituberculosis drugs in India. In: Biehl J, Petryna A. When people come first: critical issues in global health. Princeton University Press, 2013: 252–275.
44. Seeberg J. Connecting Pills and People. Med Anthropol Q. 2012;26(2):182–200.
45. Dokania AK, Dokania AK. Pharmaceutical Marketing in Rural Setting. Int J Manag Int Buisiness Stud. 2014;4(3):239–48.
46. V Yellappa, N Rao. Incentive for tuberculosis care in the private sector in India: a qualitative study. Int J Tuberc Lung Dis. Supplement 1. 2014;18(11),S251.
47. Arun Gadre, Nilangi Sardeshpande. Cut practice in the private health care. EPW. 2017;52(48).
48. Frederick A, Das M, Mehta K et al., Pharmacy based surveillance for identifying missing tuberculosis cases: A mixed methods study from South India. *Indian J TB 2*021; 68: 51-58 https://doi.org/10.1016/j.ijtb.2020.09.017

For peer review only - http://bmjopen.bmj.com/site/about/guidelines.xhtml

49. Miller, Das & Pai. 2018. Quality of tuberculosis care by Indian pharmacies: Mystery clients offer new insights. https://doi.org/10.1016/j.jctube.2017.11.002
50. Farooqui et al., 2020. The impact of stringent prescription-only antimicrobial sale regulation (Schedule H1) in India: an interrupted time series analysis, 2008–18, JAC-Antimicrobial Resistance, Volume 2, Issue 3, September 2020, dlaa076, https://doi.org/10.1093/jacamr/dlaa076
51. Chadalavada V, Babu SM, Balamurugan K. Nonprescription sale of schedule H1 antibiotics in a city of South India. *Indian J Pharmacol*. 2020;52(6):482-487. doi:10.4103/ijp.IJP_244_19
52. Sanchi Shah, Shimoni Shah, Sheela Rangan.etal. Effect of public-private interface agency in Patna and Mumbai, India: Does it alter durations and delays in care seeking for drug-sensitive pulmonary tuberculosis? Gates Open Research 2020, 4:32
53. Second Round of Impact Grant. Website of WHO-TDR: http://www.who.int/tdr/news/2015/impact-grant/en/.

For peer review only

For peer review only - http://bmjopen.bmj.com/site/about/guidelines.xhtml

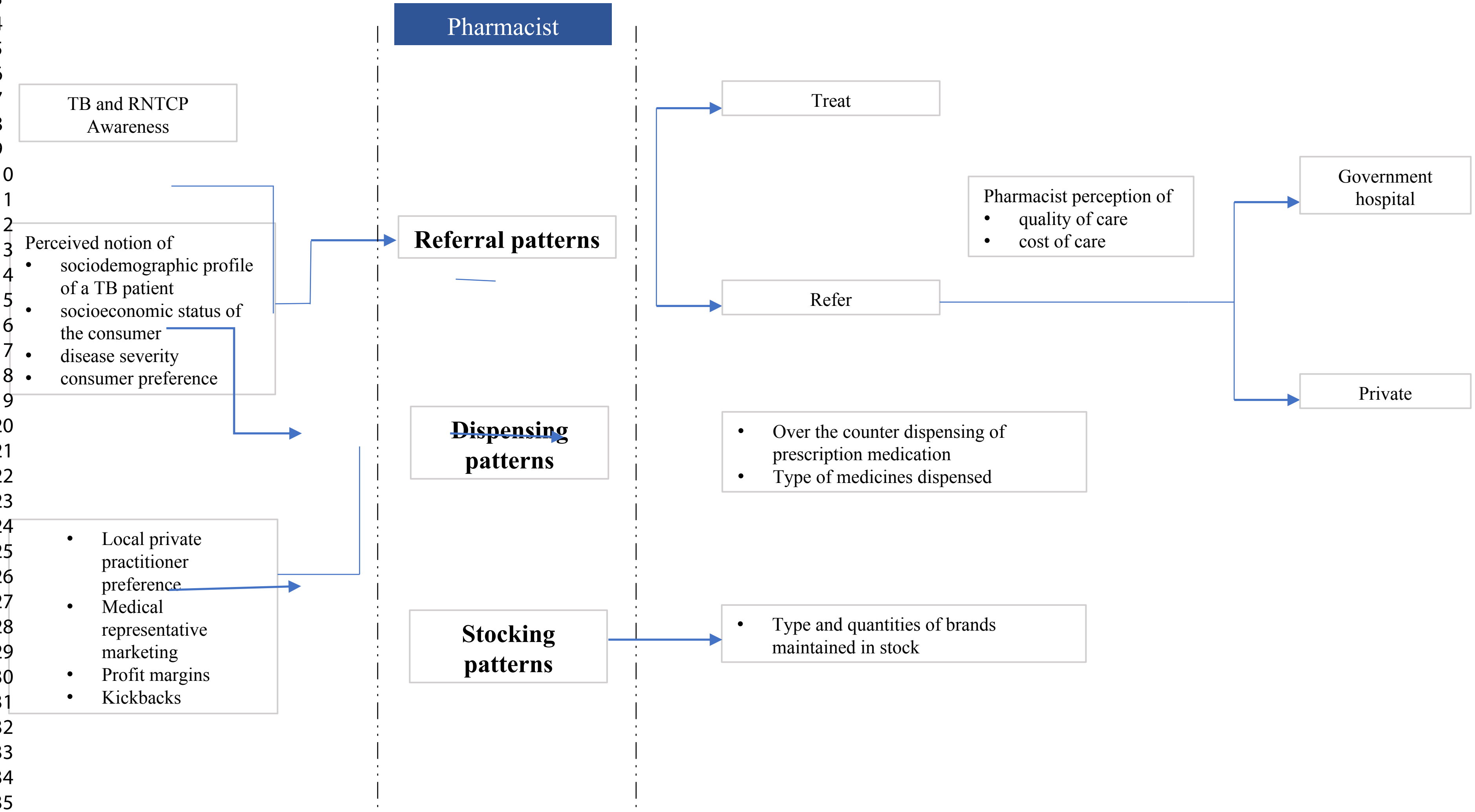
Pharmacist
TB and RNTCP Awareness
Perceived notion of
• sociodemographic profile of a TB patient
• socioeconomic status of the consumer
• disease severity
• consumer preference
• Local private practitioner preference
• Medical representative marketing
• Profit margins
• Kickbacks
Referral patterns
Dispensing patterns
Stocking patterns
Treat
Refer
Pharmacist perception of
• quality of care
• cost of care
Government hospital
Private
• Over the counter dispensing of prescription medication
• Type of medicines dispensed
• Type and quantities of brands maintained in stock

Standards for Reporting Qualitative Research (SRQR)*

http://www.equator-network.org/reporting-guidelines/srqr/

Title and abstract

	Page/line no(s).
**Title** - Concise description of the nature and topic of the study Identifying the study as qualitative or indicating the approach (e.g., ethnography, grounded theory) or data collection methods (e.g., interview, focus group) is recommended	page 1/
**Abstract** - Summary of key elements of the study using the abstract format of the intended publication; typically includes background, purpose, methods, results, and conclusions	page 1

Introduction

**Problem formulation** - Description and significance of the problem/phenomenon studied; review of relevant theory and empirical work; problem statement	page 1 & 2
**Purpose or research question** - Purpose of the study and specific objectives or questions	page 2/line 13-22

Methods

**Qualitative approach and research paradigm** - Qualitative approach (e.g., ethnography, grounded theory, case study, phenomenology, narrative research) and guiding theory if appropriate; identifying the research paradigm (e.g., postpositivist, constructivist/ interpretivist) is also recommended; rationale**	page 4/lines 22-32
**Researcher characteristics and reflexivity** - Researchers' characteristics that may influence the research, including personal attributes, qualifications/experience, relationship with participants, assumptions, and/or presuppositions; potential or actual interaction between researchers' characteristics and the research questions, approach, methods, results, and/or transferability	page 4
**Context** - Setting/site and salient contextual factors; rationale**	page 3/lines 25-43
**Sampling strategy** - How and why research participants, documents, or events were selected; criteria for deciding when no further sampling was necessary (e.g., sampling saturation); rationale**	page 4/lines 2-9
**Ethical issues pertaining to human subjects** - Documentation of approval by an appropriate ethics review board and participant consent, or explanation for lack thereof; other confidentiality and data security issues	page 11/ lines 23-30
**Data collection methods** - Types of data collected; details of data collection procedures including (as appropriate) start and stop dates of data collection and analysis, iterative process, triangulation of sources/methods, and modification of procedures in response to evolving study findings; rationale**	page 4/lines 11-20

For peer review only - http://bmjopen.bmj.com/site/about/guidelines.xhtml

**Data collection instruments and technologies** - Description of instruments (e.g., interview guides, questionnaires) and devices (e.g., audio recorders) used for data collection; if/how the instrument(s) changed over the course of the study	page 4/lines 11-20
**Units of study** - Number and relevant characteristics of participants, documents, or events included in the study; level of participation (could be reported in results)	page 4/lines 2-9
**Data processing** - Methods for processing data prior to and during analysis, including transcription, data entry, data management and security, verification of data integrity, data coding, and anonymization/de-identification of excerpts	page4/lines 14-20
**Data analysis** - Process by which inferences, themes, etc., were identified and developed, including the researchers involved in data analysis; usually references a specific paradigm or approach; rationale**	page 4/21-33
**Techniques to enhance trustworthiness** - Techniques to enhance trustworthiness and credibility of data analysis (e.g., member checking, audit trail, triangulation); rationale**	page 4/lines 33-35

Results/findings

**Synthesis and interpretation** - Main findings (e.g., interpretations, inferences, and themes); might include development of a theory or model, or integration with prior research or theory	page 6-8
**Links to empirical data** - Evidence (e.g., quotes, field notes, text excerpts, photographs) to substantiate analytic findings	page 6-8

Discussion

**Integration with prior work, implications, transferability, and contribution(s) to the field -** Short summary of main findings; explanation of how findings and conclusions connect to, support, elaborate on, or challenge conclusions of earlier scholarship; discussion of scope of application/generalizability; identification of unique contribution(s) to scholarship in a discipline or field	page 9-10
**Limitations** - Trustworthiness and limitations of findings	page 10

Other

**Conflicts of interest** - Potential sources of influence or perceived influence on study conduct and conclusions; how these were managed	page 11/line 22
**Funding** - Sources of funding and other support; role of funders in data collection, interpretation, and reporting	page 11/line 20

*The authors created the SRQR by searching the literature to identify guidelines, reporting standards, and critical appraisal criteria for qualitative research; reviewing the reference lists of retrieved sources; and contacting experts to gain feedback. The SRQR aims to improve the transparency of all aspects of qualitative research by providing clear standards for reporting qualitative research.

For peer review only

For peer review only - http://bmjopen.bmj.com/site/about/guidelines.xhtml

**The rationale should briefly discuss the justification for choosing that theory, approach, method, or technique rather than other options available, the assumptions and limitations implicit in those choices, and how those choices influence study conclusions and transferability. As appropriate, the rationale for several items might be discussed together.

Reference:

O'Brien BC, Harris IB, Beckman TJ, Reed DA, Cook DA. **Standards for reporting qualitative research: a synthesis of recommendations.** *Academic Medicine*, Vol. 89, No. 9 / Sept 2014 DOI: 10.1097/ACM.0000000000000388

For peer review only

For peer review only - http://bmjopen.bmj.com/site/about/guidelines.xhtml

BMJ Open

Understanding Dynamics of Private Tuberculosis Pharmacy Market: A Qualitative Inquiry from a South Indian district

Journal:	*BMJ Open*
Manuscript ID	bmjopen-2021-052319.R2
Article Type:	Original research
Date Submitted by the Author:	27-Dec-2021
Complete List of Authors:	Yellappa, Vijayashree; Institute of Public Health Bengaluru, Health service delivery<br>Bindu, Himabindu; Institute of Public Health Bengaluru, Public Health<br>Rao, Neethi; Institute of Public Health Bengaluru, Health service<br>Narayanan, Devadasan; Institute of Public Health Bengaluru, Health service
**Primary Subject Heading**:	Public health
Secondary Subject Heading:	Health policy, Health services research, Global health, Public health, Qualitative research
Keywords:	Tuberculosis < INFECTIOUS DISEASES, Health policy < HEALTH SERVICES ADMINISTRATION & MANAGEMENT, Organisation of health services < HEALTH SERVICES ADMINISTRATION & MANAGEMENT, Public health < INFECTIOUS DISEASES, PRIMARY CARE

SCHOLARONE™
Manuscripts

BMJ

I, the Submitting Author has the right to grant and does grant on behalf of all authors of the Work (as defined in the below author licence), an exclusive licence and/or a non-exclusive licence for contributions from authors who are: i) UK Crown employees; ii) where BMJ has agreed a CC-BY licence shall apply, and/or iii) in accordance with the terms applicable for US Federal Government officers or employees acting as part of their official duties; on a worldwide, perpetual, irrevocable, royalty-free basis to BMJ Publishing Group Ltd ("BMJ") its licensees and where the relevant Journal is co-owned by BMJ to the co-owners of the Journal, to publish the Work in this journal and any other BMJ products and to exploit all rights, as set out in our licence.

The Submitting Author accepts and understands that any supply made under these terms is made by BMJ to the Submitting Author unless you are acting as an employee on behalf of your employer or a postgraduate student of an affiliated institution which is paying any applicable article publishing charge ("APC") for Open Access articles. Where the Submitting Author wishes to make the Work available on an Open Access basis (and intends to pay the relevant APC), the terms of reuse of such Open Access shall be governed by a Creative Commons licence – details of these licences and which Creative Commons licence will apply to this Work are set out in our licence referred to above.

Other than as permitted in any relevant BMJ Author's Self Archiving Policies, I confirm this Work has not been accepted for publication elsewhere, is not being considered for publication elsewhere and does not duplicate material already published. I confirm all authors consent to publication of this Work and authorise the granting of this licence.

For peer review only

Understanding Dynamics of Private Tuberculosis Pharmacy Market: A Qualitative Inquiry from a South Indian district

Yellappa Vijayashree[1,] *Himabindu BL[1], Neethi Rao,[1] Narayanan Devadasan[1]*

Affiliations: [1] Institute of Public Health, Bangalore

Corresponding author
Vijayashree Yellappa
Adjunct Faculty, Institute of Public Health
3009, II-A Main, 17th Cross, Krishna Rajendra Rd,
Banashankari Stage II, Bengaluru, Karnataka 560070
India
Telephone: 91-9008601074
Email: vijayashreehy4@gmail.com

Ethical issues: Study received the approval from the institutional ethics committee of Institute of public health, Bangalore.

Abstract

Objectives: in India, Retail private pharmacists (RPPs) are often patients' first point of contact including Tuberculosis (TB). We assessed the factors influencing RPP's referral of patients with chest symptoms to the National TB elimination programme (NTEP) and the way business is carried out with reference to TB drugs.

Design: We conducted semi-structured interviews with a purposive sample of 41 RPPs in a south Indian district between May to October 2013. Data was collected from urban area (21 RPPs) and rural area (20 RPPs) employing the principle of data saturation. Data were analysed thematically using NVivo 9.

Results: Knowledge and compliance of RPPs regarding TB symptoms and regulatory requirements were found to be poor. The RPPs routinely dispensed medicines over-the-counter and less than half of the respondents had pharmacy qualifications. None of them had received TB related training, yet half of them knew about TB symptoms. Practice of self-referrals was common particularly among economically poorer populations who preferred purchasing medicines over-the-counter based on RPP's advice. TB patients' inability to purchase the full course of TB drugs was conspicuous. Rural RPPs were more likely to refer patients with TB symptoms to the NTEP compared to urban ones who mostly referred such clients to private practitioners (PPs). Reciprocal relationships between the RPPs, PPs, medical representatives and the prevalence of kickbacks influenced RPP's drug stocking patterns. PPs wielded power in this nexus, especially in urban areas.

Conclusion: India hopes to end TB by 2025. Our study findings will help the NTEP to design policy and interventions to engage RPPs in public health initiatives by taking cognisance of symbiotic relationships and power differentials that exist between PPs, RPPs and medical representatives. Concurrently, there should be a strong enforcement mechanism for existing regulatory norms regarding over-the-counter sales and record keeping.

For peer review only - http://bmjopen.bmj.com/site/about/guidelines.xhtml

Key words: pharmacists, private practitioners, RNTCP, NTEP, Tuberculosis, Public Private Mix, kickbacks, on-counter sale, PPM, India

Word count: 4042

Strengths and limitations of the study

- Work was undertaken to study the phenomenon naturalistically in the implementation setting, thereby providing sound empirical evidence on RPP's practices.
- Study has employed a rigorous qualitative research approach that revealed the reciprocal relationships between RPPs, PPs and medical representatives influencing RPP's drugs stocking patterns.
- Some of the initially selected pharmacists refused interviews so the data available from this study may not be fully representative of the community.
- The study draws findings based on self-reporting by RPPs, which may not necessarily correspond to their actual practice.
- We considered only RPPs registered with district drug controller, wherein many non-registered pharmacies also dispense drugs over the counter.

Introduction

Tuberculosis (TB) is the leading infectious killer globally. Ten countries accounted for 75% of the cases, wherein India and China accounted for 39% of the global gap[1]. Though Government of India offers free quality assured TB diagnosis and treatment through National TB Elimination Programme (NTEP), which was earlier known as Revised National TB Control Program[2], more than half of TB cases are managed by private practitioners (PPs) in the country[3,4]. Evidence shows that PPs rarely follow standard TB management guidelines[5,6] and thus pose a threat of severe forms of drug resistant TB.

In most parts of Asia, retail private pharmacists (RPPs) are often patients' first point of contact with the health care system[7,8] and they tend to dispense cough syrups, antibiotics, anti-allergic medicines to patients with chronic cough without physician prescription and rarely refer them for TB testing[9,10]. India has about 630766 RPPs constituting an important part of the private health sector[11] and for many patients, pharmacies may be their first point of contact, where most drugs including antibiotics, can be purchased over-the-counter[12]. Studies have found that 83% of the surveyed RPPs received up to five prescriptions of anti-TB drugs weekly[13], a finding reinforced by other study which assessed the size and characteristics of private sector anti TB drug sales in India[14].

Evidence shows that chest symptomatic who sought care from RPPs at the first instance are more likely to have long diagnostic delays[15]. Nearly half of the RPPs do not refer chest symptomatic and thus contribute to delays in diagnosis and treatment[12]. Early diagnosis and treatment initiation are crucial to break the chain of transmission of TB in the community. Delays in the diagnosis increase the chances of complications and mortality. It is therefore argued that RPPs could play an important role in the early detection of TB cases by facilitating patient pathways to TB care[16,17], but it is not the case now.

For peer review only - http://bmjopen.bmj.com/site/about/guidelines.xhtml

The NTEP has committed to providing free, high quality TB care to patients managed in private health sector through public private mix (PPM) strategy[18,19]. In 2012, the concept of PPM was expanded to RPPs after successful pilots in Mumbai in collaboration with Indian pharmaceutical association. Presently the governments are expected to train RPPs to identify and refer chest symptomatic to the NTEP and provide directly observed treatment short course[20]. RPPs receive no payment for providing such referral services. Engagement of RPPs is important not only to improve TB detection and care, but also limit the abuse of antibiotics. With this background, in 2013 the government introduced the Schedule H1 as an amendment to the Drugs and Cosmetics Rule of 1945, with the intent to control rampant misuse of antibiotics through over the counter dispensing[21].This mandates the chemist to maintain a separate register where identity of the patient, contact details of the prescribing doctor and the dispensed quantity of the drug are to be recorded and maintained for at least 3 years. Further, TB was made a notifiable disease in 2012, which mandated private health players to notify TB patients either diagnosed or treated in private sector[22].

There have been studies investigating the potential of RPPs to contribute to TB care[23,24,25.] Attempts at involving RPPs particularly in TB control activities have not always been successful. There is dearth of literature from India on the potential of RPPs to contribute to TB control activities. To develop appropriate interventions, it is essential to understand factors that influence RPP's behaviour and how this could be changed to engage RPPs in TB control activities. With this background, we undertook a study to assess (i) RPP's referral practices linked to NTEP (ii) stocking and dispensing patterns of anti -TB drugs (iii) clients' TB drugs purchasing patterns and (iv) explore the provision of kickbacks to RPPs, if any.

This study was conducted as part of a larger research project to evaluate the results-based financing strategies for TB control in India.

Methods

Study Setting

Study was carried out in two sites in Karnataka state, India, For the rural setting, Tumkur district (population of 2.8 million) was considered since 80% of the population in the district resides in villages. For the urban setting, Tumkur city (head quarter of Tumkur district, population of 302,143) and KG Halli (population 44,000), one of the 198 administrative wards of Bangalore city was selected.

Study settings consist of both private and public health facilities. TB services under the NTEP are provided free of cost through government facilities. Structure and functioning of the NTEP is elaborated elsewhere[26]. Patients can avail NTEP services either directly accessing public health facilities or through referrals by PPs/RPPs.

Retail private pharmacies are privately owned and they sell drugs for profit, paid out-of-pocket by the clients. These pharmacies range from high-end big outlets staffed by qualified pharmacists to small, roadside stalls staffed by personnel without formal qualifications by utilizing the license of pharmacists who lent their certificates for money. According to regulations, minimum qualification for registration as a pharmacist is either diploma or degree in pharmacy from an institution approved by pharmacy council of India[27]. There are typically two types of private pharmacies; 'attached' are the ones which are attached to a private health facility and 'stand-alone' with no attachment to a health facility. Patients

For peer review only - http://bmjopen.bmj.com/site/about/guidelines.xhtml

directly buy medicines from these pharmacies over-the counter with or without a valid prescription.

Study participants and sampling

We targeted 40 semi-structured interviews purposively with RPPs, 20 each from rural and urban settings, applying the principle of data saturation. In Tumkur district, we randomly selected RPPs from the list maintained with district drug controller. In KG Halli, we considered all 44 pharmacies that were identified through census. Including both study sites, total of 77 pharmacies were visited. During the visits, 14 pharmacies were closed and 23 RPPs refused to participate in the study. Overall a total of 41 RPPs participated in the study (20 from rural and 21 from urban setting; 14 from KG halli and seven from Tumkur city). All except three pharmacies included in study were stand-alone stores.

Data Collection

Data collection happened May to October 2013. Semi-structured interviews were conducted with a staff who dispensed drugs in pharmacies, irrespective of their qualification. The topic guide covered RPP's referral practices linked with NTEP, stocking and dispensing of TB drugs, clients' TB drugs purchasing patterns and provision of kickbacks. Interview guide was translated to local language, Kannada and pilot tested before conducting the actual interviews. Information brochure was shared with participants and the objectives of the research was explained. An appointment was sought and interviews were conducted in the vicinity of pharmacies. Duration of interviews ranged from 30 to 45 minutes. All interviews were digitally recorded except four participants who refused audio recording (all from urban setting). Detailed notes were recorded from such interviews.

Data analysis

Audio-recorded interviews were translated into English and transcribed verbatim by professional transcribers. Data was managed and analysed with the support of QSR NVivo 9. We conducted a thematic analysis[28,29]. We combined deductive and inductive approaches to analyse the data. The deductive approach was based on the research questions and new themes emerging from the data were included (inductive approach)[30]. Significant statements relating to the factors influencing RPP's TB management practices were identified as basic codes. VY, HB and NR devised a coding scheme jointly and this coding scheme was tested on a handful of interviews. These initial codes were then refined and organised at a broader conceptual level into themes by grouping them together[28]. Final coding framework is shown in the Table 1. In the later stages of data analysis, we explored relationships between the themes, across different categories of participants to identify patterns in the data. To increase the internal validity of the analysis, the coding scheme, the memos and the emerging themes were regularly discussed among the authors. Figure 1 represents the different themes emerged from the data.

Table 1: Coding Framework

**Initial coding framework**	**Final coding framework**
Qualification, age and number of years of business	**Study participants characteristics**

For peer review only - http://bmjopen.bmj.com/site/about/guidelines.xhtml

Number of health facilities present in the catchment area<br>Clients load<br>Common ailments for which drugs are dispensed on counter<br>Membership and participation in the pharmacy association	
Perception of TB<br>Awareness about the NTEP<br>Whether undergone TB training ?If, yes, where and when<br>Awareness about TB notification and other regulatory norms	**RPP's awareness about TB and** NTEP
Factors that influence client's self-referrals to seek care from RPPs<br>Type of drugs sold over counter for chest symptoms<br>RPP's response to self-referred chest symptomatic<br>Factors influencing RPP's referrals of chest symptomatic to health providers and the NTEP	**RPP's practices linked to chest symptomatic**
Type of drugs prescribed by private practitioners for chest symptoms<br>Prescription patterns of TB drugs by PPs<br>Factors influencing RPP's stocking of TB drugs	**RPP's stocking patterns of TB drugs**
Profile of TB patients<br>Perception of problems faced by TB patients<br>Cost of TB drugs to patients<br>Factors influencing patient's purchasing of TB drugs<br>Factors influencing patient's choice of pharmacies	**Clients' drugs purchasing patterns**
Profile of the health providers who manage TB patients<br>Factors determining the health provider's choice of pharmacies and use of TB drugs<br>Provision of kickbacks to referring private practitioners	**Provision of Kickbacks to private practitioners**

Patient and public involvement: Patients and the public were not involved in the design, implementation, analysis or dissemination of the study.

Results

Participant's characteristics

Average age of study participants was 42 years. Only 18(43%) of the participants had pharmacy qualification and others were graduates in other disciplines. Pharmacies were open for at least 12 hours starting from 9.00 am to 9.00 pm. Rural pharmacies had less number of PPs in the catchment area compared to urban ones, which were seemingly crowded. Nearly all RPPs were aware of professional pharmacists' associations, but only 20% of the qualified

RPPs were member of these associations. Details of RPPs characteristics are provided in table 2.

Table 2- Demographic data of the RPPs

Characteristic	Rural	Urban
	Gender	
Male	22	16
Female	1	2
	Age	
18-29	6	3
30-49	15	10
50 and above	2	4
	Number of years working at the pharmacy	
0-5	4	4
6-10	9	9
11-20	6	3
>20	2	0
Not available	2	0
	Approximate number of customers per day	
0-50	8	8
51-100	8	6
>100	3	2
Not available	4	1
	Approximate number of patients with complaint of cough per day	
0	1	1
1-15	5	9
16-30	2	1
>30	0	1
Not available	15	5

***RPP's awareness about TB and the** NTEP*

Almost all RPPs perceived that incidence of TB is coming down and it is no longer a problem in the community. None of the respondents had received any TB related training from the NTEP, yet half of them knew about general symptoms and mode of spread of TB. Major source of information was friends, mass media and billboards. Twelve RPPs (29%) had no idea about TB. Most RPPs knew about the NTEP, but only one RPP from the rural setting reported to have received information directly from the programme. Seven RPPs from the rural setting were aware of TB notification and they considered referrals to government hospitals to be the extent of their obligations. None of the RPPs had maintained any kind of records for dispensing TB drugs.

RPP's practices in managing patients with chest symptoms

For peer review only - http://bmjopen.bmj.com/site/about/guidelines.xhtml

RPPs reported seeing around three to four chest symptomatic per day, except six urban RPPs who reported seeing more than 20 chest symptomatic per day. Half of the respondents, mostly from urban setting reported that they do not dispense drugs without valid prescriptions. A quote:

"If they(clients) come here directly, we do not entertain them. If they ask for medicines for small ailments, we give it. Otherwise we inform them to go to the doctor" (U_10).

Remaining half of RPPs, mostly from rural setting reported dispensing drugs over-the-counter for chest symptomatic without a prescription. They reasoned that, they do this only for patients with cold and cough as it is a common illness, as opposed to diabetes and hypertension which were considered to be serious illnesses. A narrative:

"We commonly give medicines to such people who are having cold and cough. But for Sugar and BP (hypertension), we have to give specific tablets as prescribed by doctor. Even if they (clients) ask also, we are not supposed to give like that" (R_04)

For self-refereed chest symptomatic, RPPs dispensed mix of cough syrups, anti-allergic or pain killers, out of which nine RPPs reported dispensing antibiotics to such clients. On further probing, all respondents reported that they will refer patients having cough for more than 15 days to visit nearby doctors for a thorough check-up, except one rural RPP who treated clients himself. Additionally, six respondents (four from rural and two from urban) reported that they refer such patients for sputum examination and among them two (from rural area) said they would specifically refer such cases to a government facility because of the availability of free quality assured TB diagnosis there: *"I send patients to the government hospital because the results will be good"* (R_18).

"For TB patients the government hospital will provide free medicines. So they do not come outside and purchase medicines. Only patients who have consulted a private doctor, will come for AKT4 (Anti-Koch's Treatment). *But, they are very small in number"* (R_02).

When probed, majority of the respondents indicated their interest to collaborate with NTEP, if asked for in terms of stocking DOTS drugs and referring patients for sputum examination.

RPP's stocking and dispensing of TB drugs

In total, 78% of respondents reported stocking TB drugs such as AKT3 and AKT4. Stocking of TB drugs was primarily based on the suggestions of PPs practicing in the catchment area. One RPP elaborated how PP's influence the stocking of drugs:

"They[doctors] *will send prescriptions. Otherwise they will write it as 'keep in stock' and send it. If a TB patient comes to our pharmacy and informs us that he will take medicines here continuously, then we will get that medicine" (*R_02).

Other factor influencing stocking of TB drugs was the promotion of certain brands by medical representatives to provide information on profit margins. This heavily influenced RPPs stocking choices. This phenomenon appeared to be more common and systematic in urban area where medical representatives tend to persuade PPs and RPPs to stock specific brands of TB drugs. A quote:

"*It is depending upon the doctors and medical representative's understanding. We look for two to three days. If doctor prescribes the same medicine then we decide to keep that drug"* (U_10)

For peer review only - http://bmjopen.bmj.com/site/about/guidelines.xhtml

"We decide by calculating profit margins of medicines. Local companies give us more margin than standard companies. Reps give us this information" (U_06)

Rural RPPs hesitated to share information about stocking and dispensing of TB drugs and the number of TB patients purchasing TB drugs compared to urban RPPs. They tend to refer patients to Government facilities since TB drugs are available free of cost there.

Clients' drugs purchasing patterns

Both urban and rural RPPs catered to client-load varying from 30 to 300 per day, who commonly purchased drugs for general weakness, diabetes, hypertension and respiratory tract infections. Nearly 60% of RPPs informed that, patients tend to directly visit pharmacies without consulting a doctor and others bring old prescriptions to purchase drugs over-the counter. Respondents justified the on-counter practice, as this was driven by consumer demand for fast relief of symptoms. They highlighted poverty being the key factor and patient tend to make trade-off to save money and time while seeking care. An excerpt:

"If patients go to a doctor, they have to pay consultation fee of Rs 50 to Rs 100. They are low economic class and cannot afford it. Hence they come here directly. They will get tablets and syrup for the same amount if they come here directly" (U_09)

Patients' choice of pharmacies in rural setting was based on trust, familiarity with RPPs. However in the urban setting, proximity to pharmacies, time constraint, unavailability of PPs, lack of availability of medicines in other pharmacies, relatively lower prices influenced patient's choice of pharmacy.

RPPs described TB patients as those who are in their middle age and financially poor. They estimated average cost of TB drugs per day to be 7 USD /month and could go as high as 300 USD/month if nutritional supplements, cough syrup and other antibiotics are combined together in a prescription. Some RPPs deemed the anti-TB drugs to be affordable, while an equal number of them reported TB drugs put a heavy financial strain on patients. More than 85% of respondents asserted that none of the TB patients purchased the entire course of medication at one time, instead they tend to buy drugs for few days in one go. On some occasions, patients either tend to reduce the number of drugs prescribed or purchase medicines when they have money. Urban RPPs mentioned that most patients are compelled to take loans for purchasing medicines. A quote:

"Only 30- 40% patients will buy 60% of medicines. They are mostly labour class people, doing daily wage work. They get the money only in the evening, hence they buy medicines daily" (U_05).

Provision of Kickbacks to PPs

RPPs having 70-100 clients per day mentioned about the provision of kickbacks to PPs seems to be routine in urban area, but sporadic in rural. RPPs (21%) narrated a systemic nexus that existed between RPPs, medical representatives and PPs. They estimated that PPs receive commission of about 40% from pharmaceutical companies. Few rural RPPs expressed that the provision of kickbacks to PPs is not a good practice as it has negative impact on their business.

"This is the main problem in the area. From the road till the end, 90% of the doctors are involved in this. Lot of companies are giving them some offers. If the doctor prescribes the particular medicines they get commission up to 40%" (U_06).

For peer review only - http://bmjopen.bmj.com/site/about/guidelines.xhtml

RPPs also reported alternate ways through which PPs made profit by owning a pharmacy attached to their clinics and got a big share in the profits. RPPs were unhappy about this arrangement as this damaged their business. A quote:

"Now a days doctors have their own medical shops. They write such prescriptions which are available with them only. They will not send the patients here, because they (doctors) will be earning commission of 30% to 40%. By chance if they take items from here, they will send it back (R_08)

Other way of earning kickbacks was to have an understanding with RPPs and PPPs tend to prescribe only such medicines that were available with that particular RPPs. A quote:

"There are doctors who have adjustments with pharmacists, and they compel patients should go to a specific pharmacy, where they get commissions" (R_10)

When RPPs were asked whether they directly pay kickbacks to PPs, all respondents denied such practice. Some regarded it as unethical with a negative impact on their reputation.

Limitations of the study

Study draws findings based on self-reporting by the RPPs, which may not necessarily correspond to their actual practice. It is possible that RPPs might be sceptical about reporting actual number of TB patients due to fear of scrutiny. Alternatively, this discrepancy could be attributed to socially desirable responses by the participants. To overcome this, we used various approaches including assuring the RPPs of anonymity and confidentiality of the information collected, indirect questioning, probing where RPPs were not particularly forthcoming with the information, amongst others. However, we acknowledge the potential for eliciting politically correct response exists and therefore the potential for over/underestimation of the extent of these issues.

Some selected pharmacists refused interviews. So the data available may not be fully representative of the community. We have included only such RPPs who are registered with district drug controller. But there are many non-registered pharmacies, who dispense drugs over the counter.

Discussion

Our study highlights the market dynamics that influence RPP's referrals patterns about presumptive TB patients and stocking patterns of anti-TB drugs. Study findings add value to the knowledge on the strategies to involve RPPs in the NTEP. Our study showed only 43% of the respondents had pharmacy qualification and none of them had received any TB related training. RPPs reported they were not aware about regulatory requirements related to TB, thus the obligatory records and registers required from government were not maintained. Though RPPs were aware of professional pharmacists' associations, only 20% of the qualified RPPs were member of this association. RPPs reported 'self-referrals' were common among patients from economically poorer section, who preferred to purchase drugs over-the-counter based on RPP's advice. Majority of RPPs referred clients having cough more than 15 days to nearby PPs. Rural RPPs were more aware of the NTEP and tend to refer TB cases to there, far more compared to the urban counterparts. RPPs reported TB patients' inability to buy full course of TB treatment because of poverty. Our study demonstrates how reciprocal relationships between RPPs, PPs and medical representatives influence RPP's anti - TB drugs

For peer review only - http://bmjopen.bmj.com/site/about/guidelines.xhtml

stocking patterns. In general, PPs wielded substantial power in this nexus and received a significant kickbacks.

We found that half of the study participants did not have training related to pharmacy, a finding that supports the results from other study[31]. Hence, it is essential that personnel who dispense drugs should be particularly be targeted for public health training irrespective of their qualification. We found RPPs were willing to contribute to TB control activities if asked for, a trend reported elsewhere[32,33,34]. Therefore, a systematic policy of mapping RPPs and orienting them about the NTEP services might prove useful in timely detection of TB cases. Professional associations play a vital role in building the capacity of RPPs. However, our study found only 20% of qualified RPPs had membership with professional organisation, a finding similar to other study[35]. A policy or an incentive to encourage RPPs to join Indian Pharmaceutical Associations, wherein they could be systematically trained.

Our study supported the findings of studies which reported the practice of self-referrals for whom drugs were dispensed over-the-counter[36,37], which was more prevalent in rural areas. We found rural RPPs were more patronised than urban ones. RPPs who dispensed drugs over-the-counter justified the practice stating it is mostly driven by consumer demand for fast relief of symptoms as reported in other study[38,39]. Although some RPPs opined that TB drugs were affordable, patients' purchasing patterns revealed that even seemingly nominal charges could prove to be a heavy financial burden for some patients, confirming other study findings[40,41]. Such voluntary adjustments in drug purchasing by patients to reduce costs may aggravate the risk of drug resistance and lead to poor outcomes. Our study respondents estimated that costs of TB diagnosis and doctor's consultations costs more than the TB drugs and this could debilitate TB patients. However, data from the national surveys indicate that the majority of household out-of-pocket expenditure is on drugs, which is at variance from the perceptions of study respondents[42]. This under-estimation of the financial burden due to drug costs may influence respondents' behaviour with regard to kickbacks, drug-pricing etc.

Our study demonstrates pharmaceutical nexus operating in the private anti-TB drugs market. Opinions of pharma company representatives influenced stocking and sale of anti-TB drugs. This resonates the findings from a study that explored the intense competition within the pharmaceutical industry and the key role-played by medical representatives to influence PP's prescriptions[43,44,45]. This finding hints at the possibility exploring a business model for subsidising private anti-TB drugs with pharma industries and also training pharma company representatives about the NTEP provisions.

PPs not only influenced RPP's TB drugs stocking practices, but also wielded lot of power, by forcing RPPs to stock medicines of their choice. This supports the finding of a study from India which showed that PPs receive kickbacks from laboratories and pharmacies (30%)[46]. This points towards formulating a regulation that forbids practise of kickbacks. Government of Maharashtra has introduced a bill - Prevention of Cut Practice in Health Care Services Bill, 2017[47]. Evaluation of the effectiveness of this law would be helpful to curb the perverse incentives engendered through the practice of kickbacks.

None of the RPPs notified TB cases nor they maintained any mandatory registers in spite of regulatory requirement. Data sharing on anti-TB drugs from pharmacies in India continues to be poor[48]. There are mixed findings about the effectiveness of Schedule H1 regulation. Some studies have shown that it has minimized on counter dispensing of first-line anti-TB

For peer review only - http://bmjopen.bmj.com/site/about/guidelines.xhtml

drugs[49,50], but another study from south Indian city reported continued irrational dispensing of antibiotics by private pharmacies[51]. Even though suitable laws come into force, their enforcement remains a challenge for an already stretched public health system and drug control authorities.

RPPs are not optimally represented in the national policy discussions in spite of the role played by them as an interface. Though the National strategic plan[18] mentions about the need for engaging chemists, modalities for operationalising this vision is lacking. It is imperative that the symbiotic relations existing between PPs, medical representatives and RPPs should be closely scrutinized for any kind of engagement to meet public health goals. States of Gujarat and Maharashtra have experimented with the setting up of a private provider interface agency to facilitate engagement of RPPs in the NTEP[52]. Investing in public provider support agency[19] that respond to pharmacists' profit making needs while promoting the optimal delivery of health care services need to be prioritised.

The NTEP thus needs to adopt multipronged interventions that combine education coupled with regulatory enforcement to engage RPPs in the TB control activities. We have addressed some of these issues in an intervention study conducted in a south Indian district [53].

Acknowledgements: Authors would like to acknowledge Mr.Ramaiah and Ms.Amrutha for facilitating data collection.

Authors' contributions: VY and ND conceptualised the study. VY collected the data and developed the manuscript. VY, HB and NR coded and analysed the data. HB and NR contributed to the manuscript. All authors have read and approved the manuscript.

Funding Statement: The work was funded by World Bank: Contract No 7165612

Conflict of interest: The authors confirm that they have no competing interests.

Patient consent for publication Not required.

Ethics approval

Study received approval from the institutional ethics committee of Institute of public health, Bengaluru. After explaining the confidentiality in local language, written consent to participate in the study was obtained from 26 participants and the remaining opted for verbal consent. Authorization for audio recording the interviews was also sought. Personal details of participants were removed to ensure confidentiality during data transcription and analysis and the audio files were anonymised. The NVivo database was password protected and was only accessible to the research team.

Data sharing statement

Data availability statement Data are available upon reasonable request.

For peer review only - http://bmjopen.bmj.com/site/about/guidelines.xhtml

For peer review only

Figure 1: Different themes emerged from the data.

References

1. Global Tuberculosis Report. 2020. World Health Organisation. https://apps.who.int/iris/bitstream/handle/10665/337538/9789240016095-eng.pdf. Accessed on August 17, 2021.
2. India TB Report 2018,RNTCP Annual Status Report. Ministry of Health and Family Welfare. https://tbcindia.gov.in/showfile.php?lid=3314. Accessed April 12, 2018.
3. Kapoor SK, Raman AV, Sachdeva KS, Satyanarayana S. How did the TB patients reach DOTS services in Delhi? A study of patient treatment seeking behavior. PLoS One. 2012;7(8).
4. Satyanarayana S, Nair SA, Chadha SS, Shivashankar R, Sharma G, Yadav S, et al. From where are Tuberculosis patients accessing treatment in India? results from a cross-sectional community based survey of 30 districts. PLoS One. 2011;6(9): e24160. doi: 10.1371/journal.pone.0024160.
5. Satyanarayana S, Subbaraman R, Shete P, Gore G, Das J, Cattamanchi A, et al. Quality of tuberculosis care in India : a systematic review. Int J Tuberc Lung Dis. 2015; 19(7): 751-63. doi: 10.5588/ijtld.15.0186.
6. Deo, S., Jindal, P. & Papineni, S. Integrating Xpert MTB/RIF for TB diagnosis in the private sector: evidence from large-scale pilots in Patna and Mumbai, India. *BMC Infect Dis*.2021; 21 (123)
7. Miller R, Goodman C. Performance of retail pharmacies in low- and middle-income Asian settings: a systematic review. Health Policy Plan. 2016;31(7): 940-953.doi:10.1093/heapol/czw007.
8. Smith F. Private local pharmacies in low- and middle-income countries: a review of interventions to enhance their role in public health. Trop Med Int Health. 2009;14(3):362–72. doi: 10.1111/j.1365-3156.2009.02232.x.
9. Vu DH, Van Rein N, Cobelens FGJ, Nguyen TTH, Le VH, Brouwers JRBJ. Suspected tuberculosis case detection and referral in private pharmacies in Viet Nam. Int J Tuberc Lung Dis. 2012;16(12):1625–9.
10. Mistry N, Rangan S, Dholakia Y, Lobo E, Shah S, Patil A. Durations and Delays in Care Seeking, Diagnosis and Treatment Initiation in Uncomplicated Pulmonary Tuberculosis Patients in Mumbai, India. PLoS One. 2016 Mar;11(3):e0152287.
11. Global Health Observatory: Health Workforce Data by country.World Health Organization website. WHO. http://apps.who.int/gho/data/node.main.A1443?lang=en. Accessed September 27, 2016.
12. Revathy R, Sahu SK, Rehman T. Status of mandatory tuberculosis case notification among pharmacy outlets in urban Puducherry: A cross-sectional descriptive study. Indian J Community Med 2021;46:344-5
13. Divakaran B, Myalil JM, Sreedharan J. Devaraya Surendranath.Sale of anti-tuberculosis drugs through private pharmacies : a cross sectional study in Kerala. India Italian journal of Publci health. 2011;8(1). DOI: http://dx.doi.org/10.2427/5639.
14. Wells WA, Ge CF, Patel N, Oh T, Gardiner E, Kimerling ME. Size and Usage

For peer review only - http://bmjopen.bmj.com/site/about/guidelines.xhtml

Patterns of Private TB Drug Markets in the High Burden Countries. PLoS One. 2011;6(5):e18964. doi:10.1371/journal.pone.0018964.
15. Yellappa V, Lefvere P, Battaglioli T, Devadasan N, Van Der Styuft P. Patient Pathways to TB diagnosis and Treatment.BMC Public Health (2017) 17:635. DOI 10.1186/s12889-017-4627-7.
16. Satyanarayana S, Kwan A, Daniels B, Subbaraman R, McDowell A, Bergkvist S, et al. Use of standardised patients to assess antibiotic dispensing for tuberculosis by pharmacies in urban India: a cross-sectional study. Lancet Infect Dis. 2016 ;16(11):1261-1268.
17. Rajeswari R, Balasubramanian R, Bose MSC, Sekar L, Rahman F. Private pharmacies in tuberculosis control - A neglected link. Int J Tuberc Lung Dis. 2002;6(2):171–3.
18. National Strategic Plan to End TB in India 2020-25. Central TB Division, Ministry of Health and Family. Welfare.https://tbcindia.gov.in/index1.php?lang=1&level=2&sublinkid=5507&lid=3528. Accessed on May 2021.
19. Guidance Document on Partnerships. 2019. Revised National Tuberculosis Control Programme. Central TB Division. Ministry of Health and Family Welfare. https://tbcindia.gov.in/showfile.php?lid=3456. Accessed on October 17 2020.
20. Training Module for Community Pharmacists.2013. Central TB Division. Ministry of Health and Family Welfare. https://tbcindia.gov.in/showfile.php?lid=3154. Accessed on October 3, 2017.
21. Hazra A. Schedule H1: Hope or hype?. Indian J Pharmacol 2014;46:361-2
22. Mandatory TB notification Gazette for private practitioners, chemists and public health staff. https://tbcindia.gov.in/WriteReadData/l892s/5329920697FAQs%20on%20Mandatory%20TB%20notification%20Gazette%20English.pdf.
23. Daftary A, Satyanarayana S, Jha N, *et al.* Can community pharmacists improve tuberculosis case finding? A mixed methods intervention study in India. *BMJ Global Health* 2019;4:e001417.
24. Konduri, N., Delmotte, E. & Rutta, E. Engagement of the private pharmaceutical sector for TB control: rhetoric or reality?. *J of Pharm Policy and Pract* 10, 6 (2017).
25. Miller R, Goodman C. Quality of tuberculosis care by pharmacies in low- and middle-income countries: Gaps and opportunities. *J Clin Tuberc Other Mycobact Dis*. 2019;18:100135. Published 2019 Dec 2. doi:10.1016/j.jctube.2019.100135.
26. Yellappa V, Lefèvre P, Battaglioli T, Narayanan D, Van Der Stuyft P. Coping with tuberculosis and directly observed treatment: a qualitative study among patients from South India. BMC Health Services Research. 2016. 16:283. DOI 10.1186/s12913-016-1545-9.
27. Pharmacy council of India. http://www.pci.nic.in/.
28. Boyatzis RE. Transforming qualitative information: thematic analysis and code development. Thousand Oaks CA: Sage; 1998.
29. Mq P. Qualitative Research and Evaluation Methods. 3rd ed. Thousand Oaks CA: Sage; 2002.
30. Burnard P, Gill P, Stewart K, Treasure E, Chadwick B. Analysing and presenting

For peer review only - http://bmjopen.bmj.com/site/about/guidelines.xhtml

qualitative data. Br Dent J. 2008;204(8):429–32.

31. Basak SC, Arunkumar A KM. Community Pharmacists ' Attitudes towards Use of Medicines in Rural India : An analysis of current situation. Int Pharm J. 2002;16(2):32–5.
32. Sabde YD, Diwan V, Saraf VS, Mahadik VK, Diwan VK, De Costa A. Mapping private pharmacies and their characteristics in Ujjain district, Central India. BMC Health Serv Res. 2011;11:351.
33. Bell CA, Eang MT, Dareth M, Rothmony E, Duncan GJ, Saini B. Provider perceptions of pharmacy-initiated tuberculosis referral services in Cambodia, 2005-2010. Int J Tuberc Lung Dis. 2012;16(8):1086–91.
34. Gharat MS, Bell CA, Ambe GT, Bell JS. Engaging community pharmacists as partners in tuberculosis control: a case study from Mumbai, India. Res Soc Adm Pharm. 2007;3(4):464–70.
35. Sabde YD, Diwan V, Saraf VS, Mahadik VK, Diwan VK, De Costa A. Mapping private pharmacies and their characteristics in Ujjain district, Central India. BMC Health Serv Res. 2011;11(1):351. doi.org/10.1186/1472-6963-11-351.
36. Basak SC, Sathyanarayana D. Evaluating medicines dispensing patterns at private community pharmacies in Tamilnadu, India. South Med Rev. 2010;3(2):27-31.
37. Brata C, Fisher C, Marjadi B, Schneider CR, Clifford RM. Factors influencing the current practice of self-medication consultations in Eastern Indonesian community pharmacies: a qualitative study. BMC Health Serv Res. 2016;16:179.
38. Rutta E, Tarimo A, Delmotte E, James I, Mwakisu S, Kasembe D, et al. Understanding private retail drug outlet dispenser knowledge and practices in tuberculosis care in Tanzania. Int J Tuberc Lung Dis. 2014;18(9):1108-13.
39. Susanne kaae, Janine Morgall Traulsen LSN. challenges to counseling customers at the pharmacy counter-why do they exist ? Res Soc Adm Pharm. 2012;8(3):253-7.
40. Tadayuki Tanimura, Ernesto Jaramillo, Diana Weil, Mario Raviglione and Knut Lo¨nnroth. Financial burden for tuberculosis patients in low- and middle-income countries: a systematic review. Eur Respir J 2014; 43: 1763–1775 | DOI: 10.1183/09031936.00193413.
41. John KR, Daley P, Kincler N, Oxlade O, Menzies D. Costs incurred by patients with pulmonary tuberculosis in rural India. Int J Tuberc Lung Dis. 2009; 13 (10): 1281-7.
42. Health in India. 2014. NSSO. Government of India. http://mospi.nic.in/sites/default/files/publication_reports/nss_rep574.pdf. Accessed on January 2, 2018.
43. Ecks S, Harper I. Public Private Mixes: the market for antituberculosis drugs in India. In: Biehl J, Petryna A. When people come first: critical issues in global health. Princeton University Press, 2013: 252–275.
44. Seeberg J. Connecting Pills and People. Med Anthropol Q. 2012;26(2):182–200.
45. Dokania AK, Dokania AK. Pharmaceutical Marketing in Rural Setting. Int J Manag Int Buisiness Stud. 2014;4(3):239–48.
46. V Yellappa, N Rao. Incentive for tuberculosis care in the private sector in India: a qualitative study. Int J Tuberc Lung Dis. Supplement 1. 2014;18(11),S251.

For peer review only - http://bmjopen.bmj.com/site/about/guidelines.xhtml

47. Arun Gadre, Nilangi Sardeshpande. Cut practice in the private health care. EPW. 2017;52(48).
48. Frederick A, Das M, Mehta K et al., Pharmacy based surveillance for identifying missing tuberculosis cases: A mixed methods study from South India. *Indian J TB 2*021; 68: 51-58 https://doi.org/10.1016/j.ijtb.2020.09.017
49. Miller, Das & Pai. 2018. Quality of tuberculosis care by Indian pharmacies: Mystery clients offer new insights. https://doi.org/10.1016/j.jctube.2017.11.002
50. Farooqui et al., 2020. The impact of stringent prescription-only antimicrobial sale regulation (Schedule H1) in India: an interrupted time series analysis, 2008–18, JAC-Antimicrobial Resistance, Volume 2, Issue 3, September 2020, dlaa076, https://doi.org/10.1093/jacamr/dlaa076
51. Chadalavada V, Babu SM, Balamurugan K. Nonprescription sale of schedule H1 antibiotics in a city of South India. *Indian J Pharmacol*. 2020;52(6):482-487. doi:10.4103/ijp.IJP_244_19
52. Sanchi Shah, Shimoni Shah, Sheela Rangan.etal. Effect of public-private interface agency in Patna and Mumbai, India: Does it alter durations and delays in care seeking for drug-sensitive pulmonary tuberculosis? Gates Open Research 2020, 4:32
53. Second Round of Impact Grant. Website of WHO-TDR: http://www.who.int/tdr/news/2015/impact-grant/en/.

For peer review only - http://bmjopen.bmj.com/site/about/guidelines.xhtml

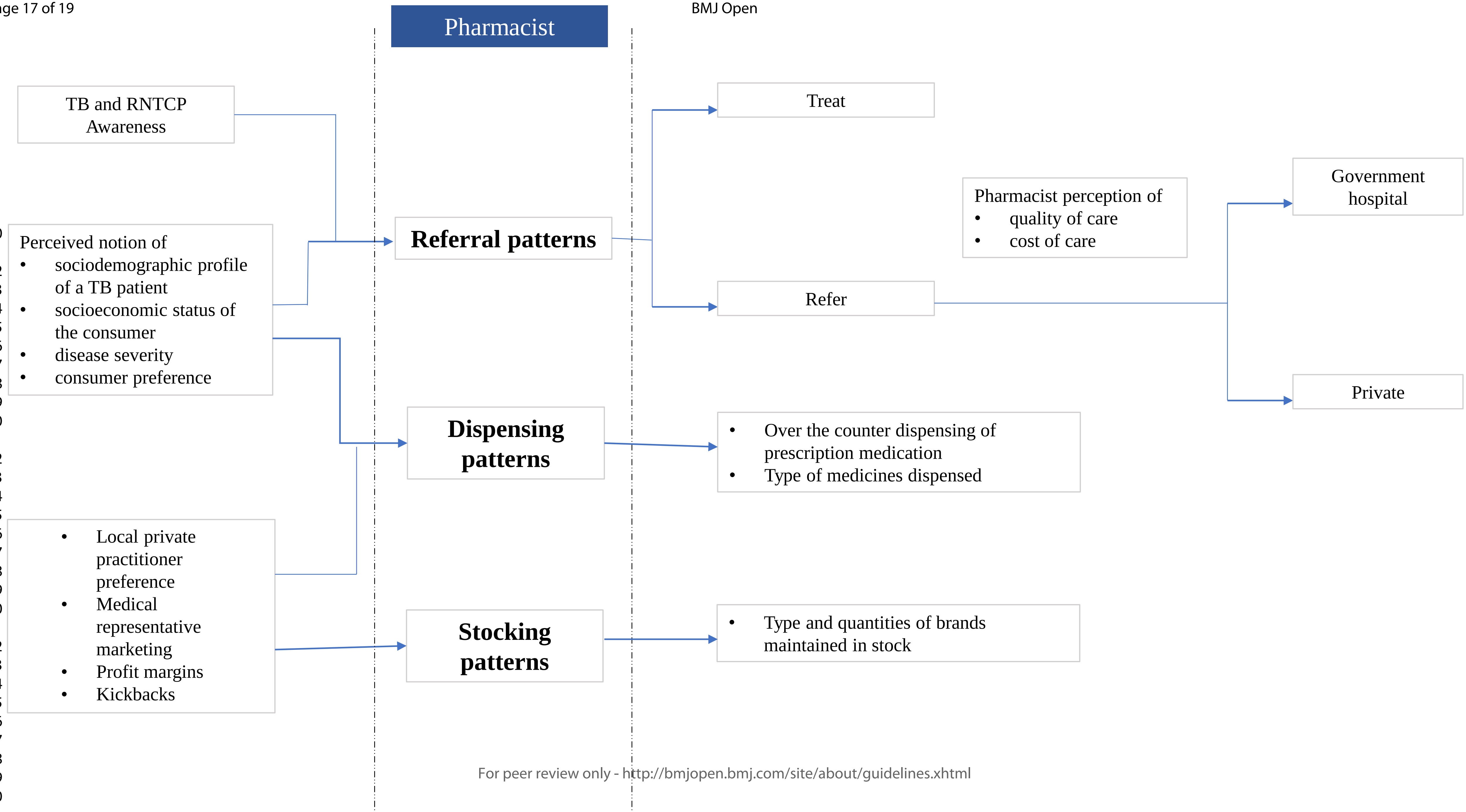
Pharmacist
TB and RNTCP Awareness
Perceived notion of
• sociodemographic profile of a TB patient
• socioeconomic status of the consumer
• disease severity
• consumer preference
• Local private practitioner preference
• Medical representative marketing
• Profit margins
• Kickbacks
Referral patterns
Dispensing patterns
Stocking patterns
Treat
Refer
Pharmacist perception of
• quality of care
• cost of care
Government hospital
Private
• Over the counter dispensing of prescription medication
• Type of medicines dispensed
• Type and quantities of brands maintained in stock

Standards for Reporting Qualitative Research (SRQR)*

http://www.equator-network.org/reporting-guidelines/srqr/

Title and abstract

	Page/line no(s).
**Title** - Concise description of the nature and topic of the study Identifying the study as qualitative or indicating the approach (e.g., ethnography, grounded theory) or data collection methods (e.g., interview, focus group) is recommended	page 1/
**Abstract** - Summary of key elements of the study using the abstract format of the intended publication; typically includes background, purpose, methods, results, and conclusions	page 1

Introduction

**Problem formulation** - Description and significance of the problem/phenomenon studied; review of relevant theory and empirical work; problem statement	page 1 & 2
**Purpose or research question** - Purpose of the study and specific objectives or questions	page 2/line 13-22

Methods

**Qualitative approach and research paradigm** - Qualitative approach (e.g., ethnography, grounded theory, case study, phenomenology, narrative research) and guiding theory if appropriate; identifying the research paradigm (e.g., postpositivist, constructivist/ interpretivist) is also recommended; rationale**	page 4/lines 22-32
**Researcher characteristics and reflexivity** - Researchers' characteristics that may influence the research, including personal attributes, qualifications/experience, relationship with participants, assumptions, and/or presuppositions; potential or actual interaction between researchers' characteristics and the research questions, approach, methods, results, and/or transferability	page 4
**Context** - Setting/site and salient contextual factors; rationale**	page 3/lines 25-43
**Sampling strategy** - How and why research participants, documents, or events were selected; criteria for deciding when no further sampling was necessary (e.g., sampling saturation); rationale**	page 4/lines 2-9
**Ethical issues pertaining to human subjects** - Documentation of approval by an appropriate ethics review board and participant consent, or explanation for lack thereof; other confidentiality and data security issues	page 11/ lines 23-30
**Data collection methods** - Types of data collected; details of data collection procedures including (as appropriate) start and stop dates of data collection and analysis, iterative process, triangulation of sources/methods, and modification of procedures in response to evolving study findings; rationale**	page 4/lines 11-20

For peer review only

**Data collection instruments and technologies** - Description of instruments (e.g., interview guides, questionnaires) and devices (e.g., audio recorders) used for data collection; if/how the instrument(s) changed over the course of the study	page 4/lines 11-20
**Units of study** - Number and relevant characteristics of participants, documents, or events included in the study; level of participation (could be reported in results)	page 4/lines 2-9
**Data processing** - Methods for processing data prior to and during analysis, including transcription, data entry, data management and security, verification of data integrity, data coding, and anonymization/de-identification of excerpts	page4/lines 14-20
**Data analysis** - Process by which inferences, themes, etc., were identified and developed, including the researchers involved in data analysis; usually references a specific paradigm or approach; rationale**	page 4/21-33
**Techniques to enhance trustworthiness** - Techniques to enhance trustworthiness and credibility of data analysis (e.g., member checking, audit trail, triangulation); rationale**	page 4/lines 33-35

Results/findings

**Synthesis and interpretation** - Main findings (e.g., interpretations, inferences, and themes); might include development of a theory or model, or integration with prior research or theory	page 6-8
**Links to empirical data** - Evidence (e.g., quotes, field notes, text excerpts, photographs) to substantiate analytic findings	page 6-8

Discussion

**Integration with prior work, implications, transferability, and contribution(s) to the field -** Short summary of main findings; explanation of how findings and conclusions connect to, support, elaborate on, or challenge conclusions of earlier scholarship; discussion of scope of application/generalizability; identification of unique contribution(s) to scholarship in a discipline or field	page 9-10
**Limitations** - Trustworthiness and limitations of findings	page 10

Other

**Conflicts of interest** - Potential sources of influence or perceived influence on study conduct and conclusions; how these were managed	page 11/line 22
**Funding** - Sources of funding and other support; role of funders in data collection, interpretation, and reporting	page 11/line 20

*The authors created the SRQR by searching the literature to identify guidelines, reporting standards, and critical appraisal criteria for qualitative research; reviewing the reference lists of retrieved sources; and contacting experts to gain feedback. The SRQR aims to improve the transparency of all aspects of qualitative research by providing clear standards for reporting qualitative research.

For peer review only

For peer review only - http://bmjopen.bmj.com/site/about/guidelines.xhtml

**The rationale should briefly discuss the justification for choosing that theory, approach, method, or technique rather than other options available, the assumptions and limitations implicit in those choices, and how those choices influence study conclusions and transferability. As appropriate, the rationale for several items might be discussed together.

Reference:

O'Brien BC, Harris IB, Beckman TJ, Reed DA, Cook DA. **Standards for reporting qualitative research: a synthesis of recommendations.** *Academic Medicine*, Vol. 89, No. 9 / Sept 2014 DOI: 10.1097/ACM.0000000000000388

For peer review only

For peer review only - http://bmjopen.bmj.com/site/about/guidelines.xhtml
